# An Integrated Workflow for Building Digital Twins of Cardiac Electromechanics—A Multi-Fidelity Approach for Personalising Active Mechanics

**DOI:** 10.3390/math10050823

**Published:** 2022-03-04

**Authors:** Alexander Jung, Matthias A. F. Gsell, Christoph M. Augustin, Gernot Plank

**Affiliations:** 1 Gottfried Schatz Research Center for Cell Signaling, Metabolism and Aging—Division of Biophysics, Medical University Graz, 8010 Graz, Austria; 2 NAWI Graz, Institute of Mathematics and Scientific Computing, University of Graz, 8010 Graz, Austria; 3 BioTechMed-Graz, 8010 Graz, Austria

**Keywords:** patient-specific modelling, human left ventricular function, cardiac mechanics, precision medicine, parameter estimation, global sensitivity analysis

## Abstract

Personalised computer models of cardiac function, referred to as cardiac digital twins, are envisioned to play an important role in clinical precision therapies of cardiovascular diseases. A major obstacle hampering clinical translation involves the significant computational costs involved in the personalisation of biophysically detailed mechanistic models that require the identification of high-dimensional parameter vectors. An important aspect to identify in electromechanics (EM) models are active mechanics parameters that govern cardiac contraction and relaxation. In this study, we present a novel, fully automated, and efficient approach for personalising biophysically detailed active mechanics models using a two-step multi-fidelity solution. In the first step, active mechanical behaviour in a given 3D EM model is represented by a purely phenomenological, low-fidelity model, which is personalised at the organ scale by calibration to clinical cavity pressure data. Then, in the second step, median traces of nodal cellular active stress, intracellular calcium concentration, and fibre stretch are generated and utilised to personalise the desired high-fidelity model at the cellular scale using a 0D model of cardiac EM. Our novel approach was tested on a cohort of seven human left ventricular (LV) EM models, created from patients treated for aortic coarctation (CoA). Goodness of fit, computational cost, and robustness of the algorithm against uncertainty in the clinical data and variations of initial guesses were evaluated. We demonstrate that our multi-fidelity approach facilitates the personalisation of a biophysically detailed active stress model within only a few (2 to 4) expensive 3D organ-scale simulations—a computational effort compatible with clinical model applications.

## 1 Introduction

Cardiovascular diseases accounted for 32% of global deaths in 2019 and remain the leading cause of death worldwide [1]. Improvements in diagnosis, therapy stratification, and planning are needed to offer personalised precision therapies that are tailored to individual patients. Computer modelling of cardiac functions show promise in this regard, owing to its unique ability of integrating disparate clinical data into a quantitative and mechanistic framework that facilitates, ultimately testing, the prediction of outcomes for various therapeutic options [2–5].

Such advanced modelling applications rely on the ability to calibrate models to clinical data, efficiently and robustly. The current, most advanced 3D multi-physics models of cardiac functions incorporate representations of electrophysiology (EP), mechanics, and haemodynamics, which cover multiple scales—from the protein up to the organ scale [6–12]. However, in most current applications, these are, at least from a functional perspective, rather generic, and do not account for the known inter-individual variability among patients [13]. While such *one-heart-fits-all* modelling approaches are useful for gaining generic mechanistic insights, they are limited in their ability to diagnose, stratify, or plan therapies on a case-by-case basis, as evidence of correspondence between model and physiological reality in a given patient is lacking. These limitations must be addressed by creating personalised models that are calibrated to clinical data. The development of techniques to create such personalised models, often referred to as digital twins [5,14], models that replicate all available observations with high accuracy, constitute a significant challenge as parameter spaces of biophysically detailed models are high-dimensional, and available clinical data are afflicted with significant observational uncertainty and residual variability [13]. Beyond the high-dimensional parameter space, many model parameters cannot be observed clinically and, thus, must be estimated indirectly, based on quantities that are clinical observable; a procedure that can become computationally expensive.

In general, calibration of 3D multi-physics models is typically attempted by calibrating, in a first step, individual physics independently, and subsequent re-calibration, to account for secondary effects due to bi-directional coupling. For models of cardiac electromechanics (EM), typically EP components are calibrated first to match activation and repolarisation, before mechanical components are calibrated to data on pressure, volume, motion, or strain. Significant improvements in the personalisation of model components have been made recently in terms of robustness, automation and fidelity, including EP [15–17], afterload [18], and passive mechanics [19,20] models. In the present study, we focus on the fully automated personalisation of active mechanics models that characterise the active mechanical behaviour through corresponding constitutive equations. These can either be described in terms of stress (active stress approach) or in terms of strain (active strain approach) [21], with the first being more commonly used. The constitutive equations of the active stress approach involve the cellular active stress that is generated in cardiomyocytes through the mechanisms of excitation–contraction coupling [22]. There are numerous models available in the literature that describe the evolution of cellular active stress with varying degrees of mechanistic detail. Purely phenomenological low-dimensional models are often preferred in clinical modelling studies. These are able to account for salient phenomena, such as length dependence underlying the Frank–Starling mechanism and are easier to constrain with the limited data available in the clinic. They are simply driven by electrical activation time to trigger the onset of contraction, whereas biophysically detailed models require space-varying intracellular calcium concentration ([*Ca* ^2+^]_i_) traces as input. For a review on active mechanics models, the interested reader is referred to Niederer et al. [3].

Several fully automated personalisation approaches have been published for purely phenomenological models [18,23–25]. Here, the number of parameters that have to be calibrated is typically small, hence, the personalisation is computationally tractable. However, an increasing number of studies, e.g., pharmacological applications [26], look explicitly at the excitation–contraction coupling mechanisms for which purely phenomenological models are not suitable. For this reason, Longobardi et al. [27] introduced a fully automated personalisation approach that can also be used for biophysically detailed models. They applied Bayesian history matching (BHM) based on Gaussian process regression (GPR) models that emulate the expensive 3D organ-scale simulations at low computational cost. While this ensures that the actual personalisation process remains inexpensive, the cost involved in the generation of training data are substantial, which poses a limitation.

The aim our study was to develop an alternative fully automated and computationally efficient approach for the personalisation of biophysically detailed active mechanics models. To this end, a two-step multi-fidelity solution is suggested in Section 2. In brief, the active mechanical behaviour in a given 3D EM model is represented by a purely phenomenological model (low-fidelity model, LFM), which is personalised at the organ scale by calibration to clinical cavity pressure data. Then, median traces of nodal cellular active stress, [*Ca* ^2+^]_i_, and fibre stretch, are generated and utilised to personalise the desired biophysically detailed model (high-fidelity model, HFM) at the cellular scale using an 0D EM model. The personalisation approach was tested for a cohort of seven patient cases for which 3D models of human LV EM have been built previously [18], see Section 2.2. To be considered viable, the personalisation approach must demonstrate sufficient robustness against clinical data uncertainty, which we show for our approach in Section 3.3. Finally, we discuss the results in Section 4 and conclude the paper with a brief summary Section 5. All required equations to implement the methods in a software framework, as well as supplementary parameter values, are given in the Appendix A.

## 2 Materials and Methods

### 2.1 Clinical Data

Clinical data of all (*N* = *7*) aortic coarctation (CoA) patients from the CARDIOPROOF cohort (NCT02591940), see [18], were used. The institutional Research Ethics Committee approved the study following the ethical guidelines of the 1975 Declaration of Helsinki. Written informed consent was attained from the patients or their guardians.

The data of each patient include anatomical 3D-whole-heart (3DWH) magnetic resonance imaging (MRI) scans, an LV volume trace obtained from short-axis cine MRI scans, and LV pressure traces of several beats obtained from invasive catheterisation. A detailed description of the data acquisition process and clinical protocols used in this study are reported in [28]. To account for inconsistencies in the LV pressure and volume traces, a three-step pre-processing was performed previously [18]: firstly, the LV volume trace was adjusted to match LV volume data points that were derived from 3DWH-MRI scans acquired during diastasis; secondly, the LV pressure traces of all beats were averaged; thirdly, the averaged LV pressure trace was synchronised with the smoothed LV volume trace. The active mechanics personalisation approach is based on biomarkers and corresponding biomarker values of the LV pressure and volume trace were obtained as described in Appendix C.

### 2.2 3d Model of Human Left Ventricular Electromechanics

#### 2.2.1 Anatomical Model

A solid model of the LV and the aortic root (AR) was created based on 3DWH-MRI scans that were acquired during diastasis. Classification tags were applied to allow for local assignments of mechanical and electrical material properties and a finite element (FE) mesh was generated (Table 1). Since the model is not in an unloaded configuration, a backward displacement method [20,29] was applied first. Then, the unloaded configuration was inflated to the configuration at the clinical end-diastolic pressure and this was deemed the reference configuration [18]. To account for the unique fibre and sheet architecture of the LV, the principal eigenaxes along the fibre direction ***f*** ^0^, the sheet direction ***s*** ^0^, and the sheet-normal direction ***n*** ^0^ in the reference configuration were assigned to each element of the LV mesh using a previously developed Laplace–Dirichlet rule-based method [30]. Details of the semi-automated workflow for building anatomical models are described in [31,32].

#### 2.2.2 Mechanical Model

The LV and the connected AR are represented by a deformable body 𝓑 that consists of particles in the configuration Ω ⊂ ℝ^3^ with Lipschitz boundary ∂Ω. The deformation from the reference configuration Ω^0^(***X***) to the current configuration Ω^*t*^(***x***) is described by the deformation gradient ***F***:= Grad ***x*** and the Jacobian *J* :=det ***F*** describes the change of the body’s volume. Furthermore, the right Cauchy-Green tensor is introduced to be ***C*** = ***F*** ^ᵀ^ ***F***. The tissue of the LV and the AR is assumed to be hyperelastic and nearly incompressible. To account for nearly incompressible behaviour, the deformation gradient is multiplicatively decomposed into the volumetric (***F***_vol_) and isochoric ( $\bar{F}$ ) parts [33]

(1) $$F_{\text{vol}}=J^{1/3}I\;\text{and}\;\bar{F}=J^{-1/3}F.$$

**Figure 1:**
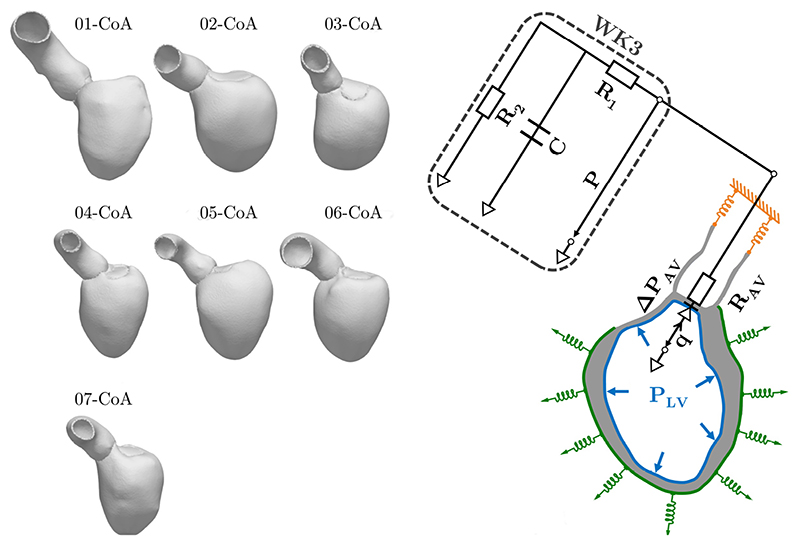
(**Left**), seven patient-specific anatomical models of the left ventricle (LV) and the aortic root from patients treated for aortic coarctation (CoA); (**right**), mechanical and afterload boundary conditions where Neumann-type pressure boundary conditions are illustrated in blue and Robin-type boundary conditions are illustrated in green (uni-directional springs that mimic the effect of the pericardium) and yellow (omni-directional springs). The mechanical model is coupled to a 0D three-element Windkessel model (WK3) that accounts for afterload conditions. Here, *R*_1_, *R*_2_ are the characteristic and peripheral resistances, respectively, and C is the arterial compliance. Pressure is denoted by *P* and the relationship between pressure and flow $q=\frac{dV}{dt}$ across the aortic valve (AV) is represented by a 0D diode model, where R_AV_ is the respective resistance. The diode model of the flow across the mitral valve and the uni-directional springs that are applied to the septum are not shown. See [18] for more details.

The spatiotemporal evolution of the displacement **𝒰** in the LV and the AR is governed by Cauchy’s first equation of motion that reads

(2) $$\rho^{0}\frac{{\text{d}}^{2}𝒰}{\text{d}t^{2}}-\text{Div}(FS)=0\;\text{in}\;\Omega^{0}\times (0,T),$$

with initial conditions

(3) $$𝒰=0\;\text{and}\;\frac{\text{d}𝒰}{\text{d}t}=0\;\text{in}\;\Omega^{0}\times \{0\},$$

where ***S*** is the second Piola–Kirchhoff stress, *ρ* ^0^ is the density in the reference configuration, $\frac{{\text{d}}^{2}𝒰}{\text{d}t^{2}}$ is the acceleration, and $\frac{\text{d}𝒰}{\text{d}t}$ is the velocity. To enforce physiological motion and to account for the cavity pressure that acts onto the endocardial surface, Robin spring boundary conditions and Neumann boundary conditions (Figure 1) are imposed on the boundaries ${\text{Γ}}_{\text{R}}^{0}$ and ${\text{Γ}}_{\text{N}}^{0}$ respectively, with $\bar{{\text{Γ}}_{\text{R}}^{0}}\cup^{\text{​}}\bar{{\text{Γ}}_{\text{N}}^{0}}=\bar{\partial {\text{Ω}}^{0}}$ .

More specifically, Robin boundary conditions refer to omni-directional springs that are applied to the aortic rim, and to uni-directional springs that are applied to the septum and to the epicardium. Latter mimics the effect of the pericardium that restricts changes of the outer shape of the heart. The pressure–volume relationship in the LV is described as in [34] and afterload is accounted for through an 0D-lumped three-element Windkessel model [35] (Figure 1) that describes the relationship between pressure and flow in the arterial system. This is coupled to the mechanical model during the ejection phase and patient-specific parameter values were already determined in previous work [18]. The relationship between pressure and flow across the aortic and mitral valve is described through a 0D diode representations (Figure 1) with forward resistances *R*_AVf_ and *R*_MVf_, and backward resistances *R*_AVb_ and *R*_MVb_, respectively. The backward resistances were set to 1000 mmHg ms mL^−1^ to prevent backflow.

Stress in the AR is assumed to arise only from the passive mechanical behaviour of the aortic tissue, i.e., ***S*** = ***S***_p_. The passive mechanical behaviour is modelled by

(4) $$S_{p}=2\frac{\partial \text{Ψ}}{\partial C},$$

where

(5) $$\text{Ψ}={\text{Ψ}}_{\text{vol}}(J)+{\text{Ψ}}_{\text{iso}}(\bar{C})$$

is the strain energy function, where $\bar{C}$ is the isochoric right Cauchy–Green tensor. The strain energy function is additively decomposed into the volumetric contribution ${\text{Ψ}}_{\text{vol}}=\frac{\kappa }{2}(J-1{)}^{2}$ with the penalty parameter *κ* and the isochoric contribution Ψ_iso_. AR tissue is assumed to be isotropic and is specified by the simple neo-Hookean strain energy function.

Stress in the LV arises not only from the passive but also from the active mechanical behaviour of ventricular tissue. Motivated by Hill’s muscle model passive and active stress are added (active stress approach) to obtain

(6) $$S=S_{\text{a}}+S_{\text{p}}.$$

The passive mechanical behaviour is modelled by Equations (4) and (5) and the volumetric contribution is ${\text{Ψ}}_{\text{vol}}=\frac{\kappa }{2}\ln(J{)}^{2}$ . LV tissue is assumed to be mechanically transversely isotropic and the isochoric contribution Ψiso is specified by the strain energy function proposed by Guccione et al. [36]. Patient-specific values for the stiffness parameter C_GUC_ were already determined in previous work [18]. Active stress is assumed to occur only in fibre direction ***f*** ^0^ and the active mechanical behaviour is thus modelled by

(7) $$S_{\text{a}}=S_{\text{a}}{\left(f^{0}\cdot Cf^{0}\right)}^{-1}f^{0}⊗f^{0},$$

where *S*_a_ is the scalar cellular active stress. This is coupled to an 0D model describing the cellular active stress evolution. Here, the purely phenomenological Tanh model [37,38] and the biophysically detailed Land model [39] are used; detailed descriptions are given in Appendices A.1 and A.2.

#### 2.2.3 Electrophysiological Model

The tissue of the LV is assumed to be electrically orthotropic and the spatiotemporal evolution of the transmembrane potential *V*_m_ is governed by the reaction-eikonal (R-E)model [40]. The eikonal equation models the spatiotemporal evolution of electrical activation (wavefronts) and reads

(8) $$\sqrt{{\left(\text{Grad}t_{\text{a}}\right)}^{⊤}v\left(\text{Grad}t_{\text{a}}\right)}=1\;\text{in}\;\Omega^{0},$$

with initial conditions

(9) $$t_{\text{a}}=t^{0}\;\text{in}\;\Gamma^{0*}\subset {\text{Ω}}^{0},$$

where ***v*** is the (wavefront) velocity tensor and *t_a_* is a positive function that gives the electrical activation (wavefront arrival) times at the locations ***X*** ∈ Ω^0^. Electrical activation is initiated at the locations ***X*** ∈ Γ^0⋆*^ in the vicinity of the septal, anterior, and posterior fascicles [34]. The development of the action potential upon electrical activation is modelled by the monodomain reaction–diffusion (R–D) equation

(10) $$\beta C_{\text{m}}\frac{\partial V_{\text{m}}}{\partial t}=\text{Div}\left(\sigma \text{Grad}V_{\text{m}}\right)+I_{\text{foot}}\left(t_{\text{a}}\right)-\beta I_{\text{ion}}\;\text{in}\;\Omega^{0}\times (0,T),$$

with initial conditions

(11) $$V_{\text{m}}=V_{{\text{m}}_{\text{res}}}\;\text{in}\;\Omega^{0}\times \{0\}.$$

The electrical activation time determines the onset of a foot current density *I*_foot_, which mimics a subthreshold electrotonic current density that initiates the action potential starting out from the resting potential V_m_res__. Velocities and conductivities ***σ*** along the three eigenaxes were set in accordance with [7].

The total ionic current density *I*_ion_ is coupled to a 0D model that describes cellular EP. This usually also integrates a model of the [*Ca* ^2+^]_i_ evolution that provides [*Ca* ^2+^]_i_ as input for biophysically detailed models of cellular active stress evolution (Appendix A.2). The mammalian ventricular cardiomyocytes model of Luo and Rudy [41], from now on referred to as the LR1 model, was used. To produce a [*Ca* ^2+^]_i_ trace that is in line with available experimental measurements in human cardiomyocytes, the purely phenomenological model of Rice et al. [42] was used and the parameter values were estimated based on experimental data reported in [43]. This model is from now on referred to as the Rice model and detailed descriptions including the parameter estimation strategy are given in Appendix A.4.

EP and mechanics are linked through various feedforward and feedback loops [44,45]. The action potential triggers active stress evolution through an increase of [*Ca* ^2+^]_i_ (electromechanical coupling) and the resulting deformation modifies the transmembrane potential (mechano-electrical coupling). The latter can be caused through stretch-activated and stretch-modified ion currents and by stretch-induced changes of [*Ca* ^2+^]_i_. Stretch-induced changes of [*Ca* ^2+^]_i_ modify not only the transmembrane potential but also the active stress (mechano-mechanical coupling). This arises from a modified affinity of troponin C for Ca^2+^ and from a modified sensitivity of the myofilaments to Ca^2+^. Electro- and mechano-mechanical coupling were considered but for the sake of simplicity, mechano-electrical coupling was not.

#### 2.2.4 Spatiotemporal Discretisation and Numerical Solution

The spatial discretisation of the mechanical and EP model equations was done based on the Galerkin finite element (FE) method using tetrahedral elements. The same average mesh resolution of approximately 690 μm was chosen for the two physics although EP processes are governed by smaller spatial scales than mechanical processes. This was made possible by the use of the R-E model that allows using a much coarser mesh resolution without causing numerical conduction slowing or block as observed in standard R-D models [40]. However, since EP processes are also governed by smaller temporal scales than mechanical processes, a different temporal discretisation was chosen. The time step for solving the EP and mechanical model equations were 0.025 ms and 1.0 ms, respectively. This choice of time step size is motivated by previous works using the same meshes [18]. To increase stability we considered Rayleigh damping of the generalised-*α* scheme used for the time integration of Equation (2), see [11]. Additionally, we made use of an approach developed by Regazzoni and Quarteroni [46] to stabilise velocity-dependent active stress models of cardiac mechanics. The cellular models always started out from their steady state computed for the clinical cardiac cycle length (duration of the clinical pressure trace; (Table 1)). If parameters were modified, the novel steady state was computed immediately. The spatial and temporal discretisation of the model and the solution of the arising systems of equations were realised in the FE framework Cardiac Arrhythmia Research Package (*CARPentry*) [40,47], built upon extensions of the openCARP EP framework [48] (http://www.opencarp.org, accessed on 30 January 2022). Numerical details for solving the EP [40,47,49] and the mechanical model equations [7] have been described in detail elsewhere. Both the solver components of the EP and the mechanical model have been verified in N-version benchmark studies [50,51]. Simulations were run on ARCHER2 (UK Research and Innovation) using 128 cores. The temporal discretisation of the cellular models and the solution of the arising systems of ordinary differential equations were realised with the tool *bench* included in openCARP. Simulations were run on a regular desktop computer using one core.

### 2.3 Active Mechanics Personalisation Approach

#### 2.3.1 Step 1: Low-Fidelity Model Personalisation at the Organ Scale

In the first step, the active mechanical behaviour in the 3D EM models is described by a LFM. This is personalised at the organ scale by minimising the difference between simulated (sim) and clinical (clin) pressure biomarker values that is obtained from available clinical cavity pressure traces. Our specific choice of the LFM is the Tanh model [37,38]. The four major parameters maximum active stress, the time constants of the contraction (rise) and relaxation (decay) phase (rise time constant), and the duration of the cellular active stress transient are active stress biomarkers that are related to corresponding pressure biomarkers. Then, the personalisation can be performed by solving the following unconstrained minimisation problem:

(12) $$\underset{p_{\text{LFM}}}{\text{min}}\overset{4}{\underset{j=1}{\sum }}{\left(\frac{{\text{B}}_{\text{P}}^{\text{clin},j}-B_{\text{P}}^{\text{sim},j}\left(p_{\text{LFM}}\right)}{B_{\text{P}}^{\text{clin},j}}\right)}^{2},$$

where *p*_LFM_ = {*S*_max_ref__, *τ_SR_*_ref_, *τ*_SD_, *t*_CR_} are the parameters of the Tanh model (Appendix A.1) and *B*_P_ = {*P*_max_, ${\frac{\text{d}P}{\text{d}t}|}_{\text{max}}$ , ${\frac{\text{d}P}{\text{d}t}|}_{\text{min}}$ , *PTD*_90_} are the pressure biomarkers (Appendix C) that are widely used, e.g., [18,26,27,37,38]). The formulation of the minimisation problems in this study is based on relative least squares as selected biomarkers vary significantly in magnitude; this would introduce an unintentional weighting of the terms otherwise. Owing to the relationship between parameters and biomarkers, a fixed point approach can be used to solve the problem with few iterations at low cost. The following choice of updates proved to be most promising for our application:

(13) $$\begin{array}{lll} S_{{\text{max}}_{\text{ref}}}^{i+1} & = & S_{{\text{max}}_{\text{ref}}}^{i}\cdot \frac{P_{\text{max}}^{\text{clin}}}{P_{\text{max}}^{\text{sim},i}},\\ \tau_{{\text{SR}}_{\text{ref}}}^{i+1} & = & \tau_{{\text{SR}}_{\text{ref}}}^{i}\cdot \frac{{\frac{\text{d}P^{\text{sim},i}}{\text{d}t}|}_{\text{max}}}{{\frac{\text{d}P\text{clin}}{\text{d}t}|}_{\text{max}}},\\ \tau_{\text{SD}}^{i+1} & = & \tau_{\text{SD}}^{i}\cdot \frac{{\frac{\text{d}P^{\text{sim},i}}{\text{d}t}|}_{\text{min}}}{{\frac{\text{d}P\text{clin}}{\text{d}t}|}_{\text{min}}},\\ t_{\text{CR}}^{i+1} & = & t_{\text{CR}}^{i}\cdot \frac{PTD_{90}^{\text{clin}}}{PTD_{90}^{\text{sim},i}}. \end{array}$$

If, additionally, a clinical cavity volume trace is available, the personalisation can be extended to valve flow models (VFM) that describe the relationship between pressure and flow across valves. In this case, the minimisation problem reads

(14) $$\underset{p_{\text{LFM}},p_{\text{VFM}}}{\text{min}}\left[\overset{4}{\underset{j=1}{\sum }}{\left(\frac{B_{\text{P}}^{\text{clin},j}-B_{\text{P}}^{\text{sim},j}\left(p_{\text{LFM}}\right)}{B_{\text{P}}^{\text{clin},j}}\right)}^{2}+\overset{n_{\text{VFM}}}{\underset{k=1}{\sum }}{\left(\frac{B_{\text{V}}^{\text{clin},k}-B_{\text{V}}^{\text{sim},k}\left(p_{\text{VFM}}\right)}{B_{\text{V}}^{\text{clin},k}}\right)}^{2}\right]$$

where *p*_VFM_ are the parameters of the VFM and *B*_V_ are related volume biomarkers. Owing to the relationship between parameters and biomarkers, a fixed point approach can be used again for solving the problem. In the simple case of a diode valve with forward resistance *R*_f_, the additional update is:

(15) $$R_{\text{f}}^{i+1}=R_{\text{f}}^{i}\cdot \frac{\tau_{\text{V}}^{\text{clin}}}{\tau_{\text{V}}^{\text{sim},i}},$$

where *B*_V_ = *τ*_V_ is the time constant of either the ejection (decay time constant *τ*_VD_) or the filling phase (rise time constant *τ*_VR_), see Appendix C.

A simulation with the current set of parameter values is performed in each iteration *i* until the minimisation problem is considered to be converged, i.e., the cost function reached a predefined threshold. Subsequently, traces of cellular active stress, [*Ca* ^2+^]_i_, and fibre stretch $(\lambda =\sqrt{f^{0}\cdot Cf^{0})}$ are extracted for each node of the LV FE grid. These traces are first aligned by the respective nodal electrical activation times and then used to generate median traces $S_{\text{a}}^{*}(t)$ , ${\left[Ca^{2+}\right]}_{\text{i}}^{*}(t)$ , and *λ* ^*^(*t*). Here, the median was chosen to be more robust against outlier traces.

In this study, the Tanh model and both the aortic and mitral VFM were personalised. These were integrated in the patient-specific 3D model of human LV EM (Section 2.2) and the required pressure and volume biomarker values were obtained from available patient-specific LV pressure and volume traces (Section 2.1). Initial guesses of the Tanh model are given in Table A1 and the forward resistances of the VFMs were initialised with 0.01 mmHg mL s ^−1^. One heart beat was simulated in each iteration and the threshold value for convergence of the minimisation problem was set to 0.1.

#### 2.3.2 Step 2: High-Fidelity Model Personalisation at the Cell Scale

**Figure 2:**
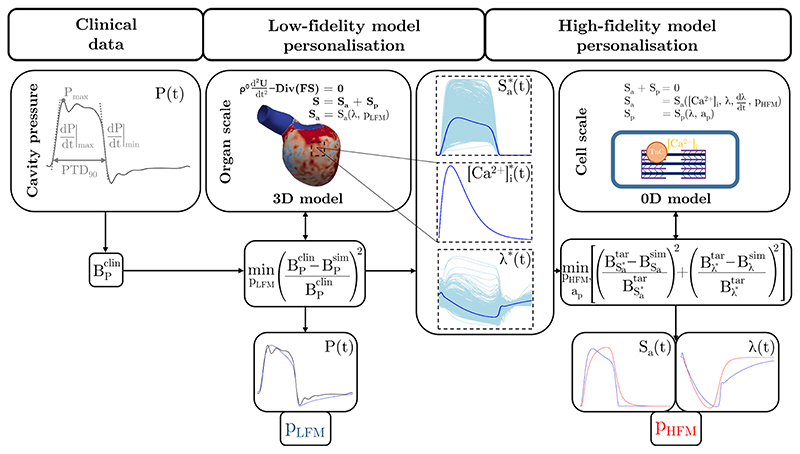
Conceptual illustration of the two-step multi-fidelity approach for personalising biophysically detailed active mechanics models. In the first step, the active mechanical behaviour in the given 3D EM model is described by a low-fidelity model (LFM). This is personalised at the organ scale by minimising the difference between simulated (sim) and clinical (clin) pressure biomarker values (B_P_) that are obtained from an available clinical cavity pressure trace. Median traces of nodal cellular active stress ( $S_{a}^{*}$ ), intracellular calcium concentration ( ${\left[Ca^{2+}\right]}_{i}^{*}$ ), and fibre stretch (λ*) are then obtained from the simulation that was produced by the personalised 3D EM models. These are utilised to personalise the high-fidelity model (HFM) at the cell scale in the second step. To this end, the HFM model is integrated in an 0D EM model that simulates the stretch of the cardiomyocyte during the cardiac cycle based on the equilibrium of active and passive stress. The median trace of nodal [*Ca* ^2+^]_i_is used as input but [*Ca* ^2+^]_i_ can also be generated from a coupled model of [*Ca* ^2+^]_i_ evolution that is integrated in the chosen cellular EP model. Personalisation is done by minimising the difference between simulated (sim) and target (tar) biomarker values that are obtained from the median traces of nodal cellular active stress ( $B_{S_{a}^{*}}$ ) and fibre stretch (B_λ*_). The parameters of the LFM and the HFM are denoted by *p*_LFM_ and *p*_HFM_, respectively. Please note that the fibre stretch in the relaxed state is 1 per definition (Section 2.3.1 and Appendix A.3).

In the second step, the median nodal traces are used to personalise the desired 0D HFM at the cell scale. Biophysically detailed models of cellular active stress evolution are functions of [*Ca* ^2+^]_i_, the fibre stretch, and the fibre stretch rate. In theory, the personalisation could be performed by using the cellular active stress trace as target and the [*Ca* ^2+^]_i_ trace, the fibre stretch trace, and the time derivative of the fibre stretch trace as input. However, the distribution of nodal fibre stretch in the myocardium at a given time point is very heterogeneous, which causes large errors in the personalisation. An exemplary distribution of nodal fibre stretch traces is shown in Figure 2. To overcome this issue, we suggest using an 0D EM models that is able to simulate the stretch of the cardiomyocyte, *λ*, during the cardiac cycle based on the equilibrium of active (*S*_a_) and passive stress (*S*_p_):

(16) $$S_{\text{a}}\left({\left[Ca^{2+}\right]}_{\text{i}},\lambda ,\frac{\text{d}\lambda }{\text{d}t},t\right)+S_{\text{p}}(\lambda ,t)=0\;\text{for}\;t\;\in \;(0,T),$$

with initial conditions

(17) $$\lambda (0)=\lambda^{0}.$$

The cellular active stress evolution is described by the HFM and the equilibrium equation can be solved in line with [46] to obtain the stretch trace and the stretch rate trace as time derivative. Here, the initial stretch *λ* ^0^ was set to the initial value of the median nodal trace and for an accurate personalisation of the active mechanics model it was found to be sufficient to match the minima *λ*_min_. To describe the cellular passive stress evolution, we used the three-element model of Land et al. [39] (Appendix A.3). Then, the minimum stretch can be controlled solely by the stiffness parameter *a*_p_. Please note that *a*_p_ may be far from physiological when interpreted in the context of single cell stiffness. This is because fibre stretch traces produced at the organ scale are not only functions of local active and passive stress but also of the existing boundary conditions (Section 2.2.2) and deformations in the vicinity (Section 2.2.3). The only purpose of *a*_p_ here is to produce a minimum stretch in line with the median nodal trace.

The personalisation can be performed by solving the following constrained minimisation problem:

(18) $$\begin{array}{ll} \underset{p_{\text{HFM}},a_{\text{p}}}{\text{min}} & \left[\overset{n_{\text{HFM}}}{\underset{j=1}{\sum }}w_{{\text{S}}_{\text{a}}}^{j}{\left(\frac{B_{{\text{S}}_{\text{a}}^{*}}^{\text{tar},j}-B_{{\text{S}}_{\text{a}}}^{\text{sim},j}\left(p_{\text{HFM}},a_{\text{p}}\right)}{B_{{\text{S}}_{\text{a}}^{*}}^{\text{tar},j}}\right)}^{2}+w_{\lambda }{\left(\frac{B_{\lambda^{*}}^{\text{tar}}-B_{\lambda }^{\text{sim}}\left(p_{\text{HFM}},a_{\text{p}}\right)}{B_{\lambda^{*}}^{\text{tar}}}\right)}^{2}\right]\\ \;\text{s}.\text{t}. & p_{\text{min}}\leq p\leq p_{\text{max}},\\ & \begin{array}{l} S_{{\text{a}}_{\text{res}}}=0,\\ 0\leq S_{\text{a}}(t),\\ {\frac{\text{d}S_{\text{a}}(t)}{\text{d}t}|}_{t_{S_{a_{\text{max}}}}}\leq 0 \end{array} \end{array}$$

where *t*_*S*_a_max___ = argmax_*t*∈[0,*T*]_ *S*_a_ (*t*), *p*_HFM_ are the parameters of the HFM, *B*_*S*_*a*__ are appropriate active stress biomarkers, and *B*_λ_* = *λ*_min_ is the stretch biomarker. The target biomarkers are obtained from the corresponding median nodal traces. In contrast to the first step, constraints are required since parameters *p* = {*p*_HFM_, *a*_p_} and the traces of cellular active stress and stretch can easily become non-physiological. The minimum requirements for the cellular active stress trace is that the resting stress is zero; that all stresses are equal or above zero; and that the stress rate is not positive after the maximum stress has been reached. If these requirements are met, no further constraints are required for the stretch trace. Non-negative weights $w_{{\text{S}}_{\text{a}}}^{j}$ and *w_λ_* were found to be helpful to control the impact of certain terms in order to obtain a physiological outcome.

Instead of using the median trace of nodal [*Ca* ^2+^]_i_ as input for the HFM, the HFM can also be coupled to a model of [*Ca* ^2+^]_i_ evolution that is integrated in the chosen cellular EP model. This comes with the advantage that the model of [*Ca* ^2+^]_i_ evolution can be included in the personalisation process. In this case, the constrained minimisation problem is extended to:

(19) $$\begin{array}{ll} \underset{p_{\text{HFM}},a_{\text{p}},p_{\text{CEM}}}{\text{min}} & \left[\overset{n_{\text{HFM}}}{\underset{j=1}{\sum }}w_{S_{\text{a}}}^{j}{\left(\frac{B_{S_{\text{a}}^{*}}^{\text{tar},j}-B_{S_{\text{a}}}^{\text{sim},j}\left(p_{\text{HFM}},a_{\text{p}},p_{\text{CEM}}\right)}{B_{S_{\text{a}}^{*}}^{\text{tar},j}}\right)}^{2}+w_{\lambda }{\left(\frac{B_{\lambda^{*}}^{\text{tar}}-B_{\lambda }^{\text{sim}}\left(p_{\text{HFM}},a_{\text{p}},p_{\text{CEM}}\right)}{B_{\lambda^{*}}^{\text{tar}}}\right)}^{2}\right],\\ \;\text{s}.\text{t}. & p_{\text{min}}\leq p\leq p_{\text{max}},\\ & S_{{\text{a}}_{\text{res}}}=0,\\ & 0\leq S_{\text{a}}(t),\\ & \frac{{\text{dS}}_{\text{a}}(t)}{\text{d}t}|{}_{t>t_{S_{{\text{a}}_{\text{max}}}}}\leq 0,\\ & 120\text{ms}\leq CTD_{50}\leq 420\text{ms},\\ & 220\text{ms}\leq CTD_{90}\leq 785\text{ms,} \end{array}$$

where *p*_CEM_ are the parameters of the [*Ca* ^2+^]_i_ evolution model. In this case, above mentioned constraints on the parameters *p* = {*p*_HFM_, *p*_CEM_, *a*_p_}and the cellular active stress trace were extended by constraints on the [*Ca* ^2+^]_i_ trace. More specifically, *CTD*_50_ and a *CTD*_90_—the [*Ca* ^2+^]_i_ transient durations at the time of 50% and 90% decay from the maximum, respectively, measured from the electrical activation time—were required to be within the range given in [43,52]. 0D cell-scale simulations are computationally much less expensive than 3D organ-scale simulations which reduced the computational cost of the personalisation approach substantially. Further improvement of computational efficiency can be achieved by reducing the number of model parameters by means of a global sensitivity analysis (GSA), see Section 2.4.

In this study, the 0D model of cellular EM consisted of the Land models of cellular active stress (Appendix A.2) and passive stress evolution (Appendix A.3), and the Rice model that describes the evolution of [*Ca* ^2+^]_i_ on a purely phenomenological basis (Appendix A.4). Both the Land model of cellular active stress evolution and the Rice model were personalised and to this end, the following active stress biomarkers were used to set up the minimisation problem: *B*_*S*_a__ = {*S*_*a*_max__, ${\frac{\text{d}S_{\text{a}}}{\text{d}t}|}_{\text{max}}$ , *STD*_30_, *STD*_50_, *STD*_90_}(Appendix C).

In addition to the stiffness parameter *a*_p_, the nine most influential parameters that were identified by means of a GSA (see Section 2.4), were considered for estimation. Initial guesses and constraints on the parameters are given in Tables A2–A5 and the weights were *w*_S_a__ = 5, 1, 5, 1, 1 and *w_λ_* = 100.

The constrained minimisation problem was solved as series of unconstrained problems by application of the penalty method (Appendix D). For solving, the population-based differential evolution method was used. This is a meta-heuristic global optimisation method [53] that was developed for multi-dimensional real-valued functions. Several studies [54,55] have demonstrated fast convergence, robustness, and good performance in real-world problems. As the gradient of the problem is not required, it can also be used for noncontinuous problems. This is advantageous for our application since some parameter value combinations within the admissible range may lead to failure of the 0D cell-scale simulation. The implementation of the differential evolution method was based on *lmfit: non-linear least-squares minimisation and curve-fitting for Python* [56] with default settings. In more detail, a Latin hypercube sampling was used to generate an initial population of candidate solutions that was then iteratively modified using the *best1bin* variant until convergence.

The two-step multi-fidelity approach is conceptually illustrated in Figure 2 and the general algorithm is given in Algorithm 1.

**Algorithm 1 Two-step multi-fidelity approach for personalising biophysically detailed active mechanics models.**

1:    Initialise counter i = 0 and parameters of the low-fidelity model $p_{\text{LFM}}^{0}$ , see Appendix A.1.

2:    **do**

3:       Solve 3D organ-scale model with low-fidelity model and parameters $p_{\text{LFM}}^{i}$ .

4:       Compute error between simulated ( $B_{\text{p}}^{\text{sim}}$ ) and clinically measured ( $B_{\text{p}}^{\text{clin }}$ ) pressure biomarkers.

5:       Update parameter set using fixed point approach, see Equations (13) and (15), to get $p_{\text{LFM}}^{i+1}$ .

6:       Update counter *i* = *i* +1.

7:    **while** error > threshold

8:    Generate median nodal traces of active stress ( $S_{\text{a}}^{*}(t)$ ), intracellular calcium concentration ( ${\left[Ca^{2+}\right]}_{\text{i}}^{*}(t)$ ), and fibre stretch (*λ* * (*t*)) from low-fidelity model results.

9:    Compute target biomarkers for active stress ( $\left(B_{S_{\text{a}}^{*}}^{\text{tar}}\right)$ ) and fibre stretch ( $\left(B_{\lambda^{*}}^{\text{tar}}\right)$ ) from $S_{\text{a}}^{*}(t)$ and λ* (*t*), respectively.

10:    Initialise counter *j* = 0 and parameters of the high-fidelity model $p_{\text{HFM}}^{0}$ , see Appendix A.2.

11:    **do**

12:       Solve low-cost 0D cell-scale model with high-fidelity model using $p_{\text{HFM}}^{j}$ and ${\left[Ca^{2+}\right]}_{\text{i}}^{*}(t)$ .

13:       Compute error between simulated ( $B_{S_{a}}^{\text{sim}}$ , $B_{\lambda }^{\text{sim}}$ ) and target ( $B_{S_{\text{a}}^{*}}^{\text{tar}}$ , $B_{\lambda^{*}}^{\text{tar}}$ ) biomarkers.

14:       Update parameter set based on differential evolution method to get $p_{\text{HFM}}^{j+1}$ .

15:       Update counter *j* = *j* + 1.

16:    **while** error > threshold

### 2.4 Global Sensitivity Analysis

The variance-based Sobol’ GSA [57,58] was used to identify the parameters that the chosen active stress biomarkers are most sensitive to in the 0D EM model. In general, when the model inputs are varied, it measures the sensitivity of the model outputs to each model input by the fraction of variance attributed to each model input alone (first-order sensitivity index S1) or by the fraction of variance attributed to each model input including interactions with the other model inputs (total-effect sensitivity index ST). The Sobol’ GSA is based on an all-at-a-time sampling strategy and the sensitivity indices range from 0 (no sensitivity) to 1 (maximum sensitivity).

In this study, the Sobol’ GSA was performed to identify the nine parameters of the Land and the Rice model (inputs) that the five active stress biomarkers (Section 2.3.2; outputs) are most sensitive to in the 0D EM model. Saltelli’s sampling scheme [59] with *N* = 1024 was applied to generate 45,506 parameter samples. Lower and upper bounds of the parameters were in line with the parameter constraints in the second step of the personalisation approach (Section 2.3.2). The simulations were performed with a cardiac cycle length of 1000 ms and to produce stretch traces in line with those seen in the organscale simulations, the initial stretch *λ* ^0^ was set to 1.1 and the stiffness factor *a*_p_ was set to 42 kPa leading to comparable minima.

Samples that produced non-physiological cellular active stress traces were excluded. The exclusion criteria were adopted from the cellular active stress constraints in the second step of the personalisation approach (Section 2.3.2) with the only difference that the resting stress was allowed to take values up to 10% of *S*_a_max__ to increase the number of data points for the analysis. Since the number of output and input values must be equal, the biomarkers of the non-physiological traces were set to the means of the biomarkers of the physiological traces.

Finally, the parameters were ranked by their sensitivity. To this end, the total-effect sensitivity indices with respect to each biomarker were added. The implementation of the GSA was based on *SALib*—*Sensitivity Analysis Library in Python* [60].

### 2.5 Robustness Analyses

#### 2.5.1 Clinical Data Uncertainty

To quantify the robustness of the active mechanics personalisation approach against uncertainties that clinical data are afflicted with, their effect on the estimated parameter values and consequences for the simulated pressure and volume traces were analysed (uncertainty propagation). First, samples of clinical biomarker values were generated that evenly filled a range of ±10% around the measured values. Then, the samples were used to perform the active mechanics personalisation.

#### 2.5.2 Initial Guess Variation

To quantify the robustness of the active mechanics personalisation approach against variation of initial guesses, their effects on the estimated parameter values, and consequences for simulated pressure and volume traces were analysed. For this purpose, samples of initial guesses that evenly filled a range of ±50% around the default values were generated. This was done for both steps.

The sampling was performed using Latin hypercube sampling implemented in *SALib* [60]. Owing to the marked computational costs involved, the robustness analyses were carried out only for the case 02-CoA and a sample size of ten and five, respectively.

## 3 Results

### 3.1 Global Sensitivity Analysis

**Figure 3:**
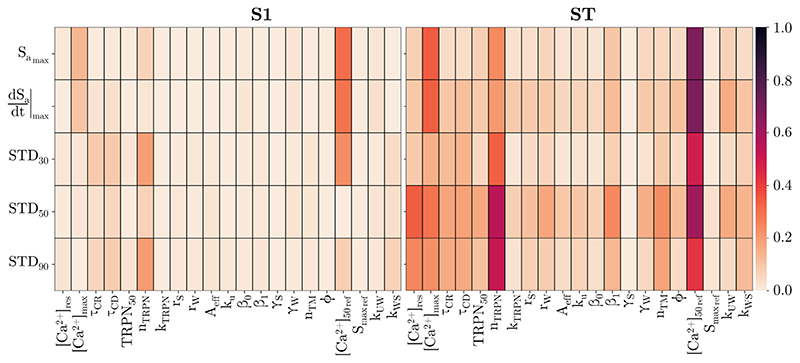
Global sensitivity of active stress biomarkers to parameters of the Land and the Rice model in the 0D EM model. The Sobol global sensitivity analysis was performed and first-order (*S*1) and total-order sensitivity indices (*ST*) are shown.

Figure 3 shows the sensitivity of the chosen active stress biomarkers to the parameters of the Land and the Rice model in the 0D EM model. The comparison of first-order (S1) and total-effect indices (ST) demonstrate that the influence of the parameters on the active stress biomarkers was primarily caused by interactions among them. For the given admissible parameter ranges, the Land model parameters [*Ca* ^2+^]_50_ref__ and *n*_TRPN_ were most influential across all biomarkers, followed by the Rice model parameters [*Ca* ^2+^]_res_ and [*Ca* ^2+^]_max_. This highlights the role of troponin C kinetics and the [*Ca* ^2+^]_i_ dynamics in the evolution of cellular active stress. The role of the [*Ca* ^2+^]_i_ dynamics is further emphasised by the fact that all parameter of the Rice model were among the nine most influential parameters: *τ*_CD_ was ranked sixth and *τ*_CR_ was ranked eight. The list was completed by the Land parameters *β*_0_, *n*_Tm_, and *k*_UW_. These nine parameters were considered in the second step of the active personalisation approach.

### 3.2 Patient Cohort Results

The active mechanics personalisation approach, as described in Section 2.3, was applied to all *N* = 7 patient cases: 01-CoA–07-CoA. In the first step, LV pressure traces were used to personalise the Tanh model and LV volume traces were used to include the aortic and the mitral VFM in the personalisation process. This was done based on the 3D model of human LV EM. For six out of seven cases, two to four iterations were needed for convergence (Table 1). For case 04-CoA, the simulation aborted at the tenth iteration and therefore, the results of the ninth iteration were taken. The convergence behaviour for this particular case is shown in Figure A2. The estimated parameter values for all models are given in Table A6.

**Figure 4:**
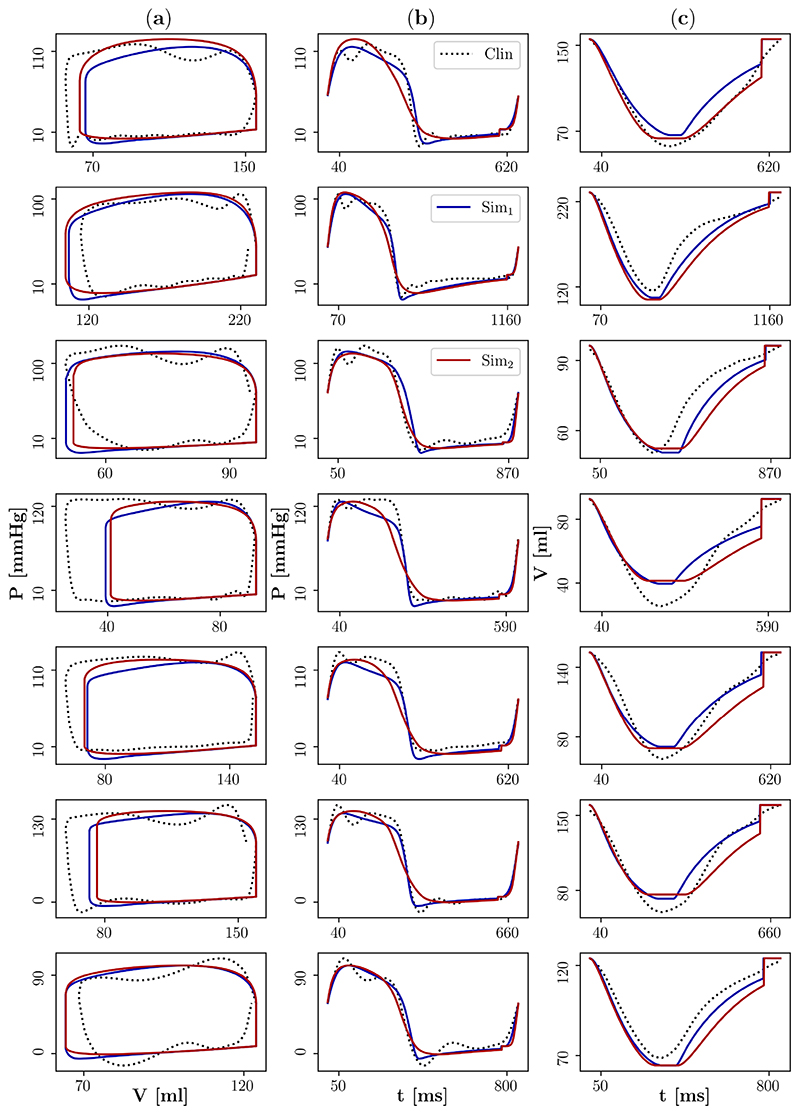
Comparison of simulated and clinical left ventricular pressure and volume after personalisation. The blue/red lines represent the simulation data Sim_1_/Sim_2_ that were produced by the 3D model of human left ventricular EM in the first/second step of the active mechanics personalisation approach. The black dots represent clinical data (Clin). (**a**) Pressure–volume loops, (**b**) pressure traces, and (**c**) volume traces are shown.

Figure 4 compares simulated and clinical LV pressure–volume loops and the individual LV pressure and volume traces. Moreover, Table 2 compares simulated and clinical values of relevant pressure and volume biomarkers (*P*_max_, ${\frac{\text{d}P}{\text{d}t}|}_{\text{max}}$ , ${\frac{\text{d}P}{\text{d}t}|}_{\text{min}}$ , *PTD*_90_, *SV*). The personalised models were able to reproduce the clinical pressure with good agreement. The relative differences between the simulated and clinical values of *P*_max_ and *PTD*_90_ had means (standard deviations; SD) of 5.4% (SD: 3.4%) and 1.4% (SD: 2.2%), respectively. For ${\frac{\text{d}P}{\text{d}t}|}_{\text{max}}$ and ${\frac{\text{d}P}{\text{d}t}|}_{\text{min}}$ , the mean relative differences were slightly larger with 13.6% (SD: 8.0%) and 7.7% (SD: 8.0%), respectively. The brief pressure drop after the first peak seen in the clinical data are considered a measurement artefact. Except for case 04-CoA, the personalised models were also able to reproduce the clinical volume with good agreement, albeit the agreement in the systolic phase was better than in the diastolic phase. The mean relative difference between the simulated and clinical *SV* was 10.0% (SD: 5.9%). However, simulated end-diastolic volumes were substantially smaller than clinical end-diastolic volumes. Except for case 04-CoA, the good agreement between simulated and clinical pressure and volume translated into a good agreement in the pressure–volume relationship.

**Figure 5:**
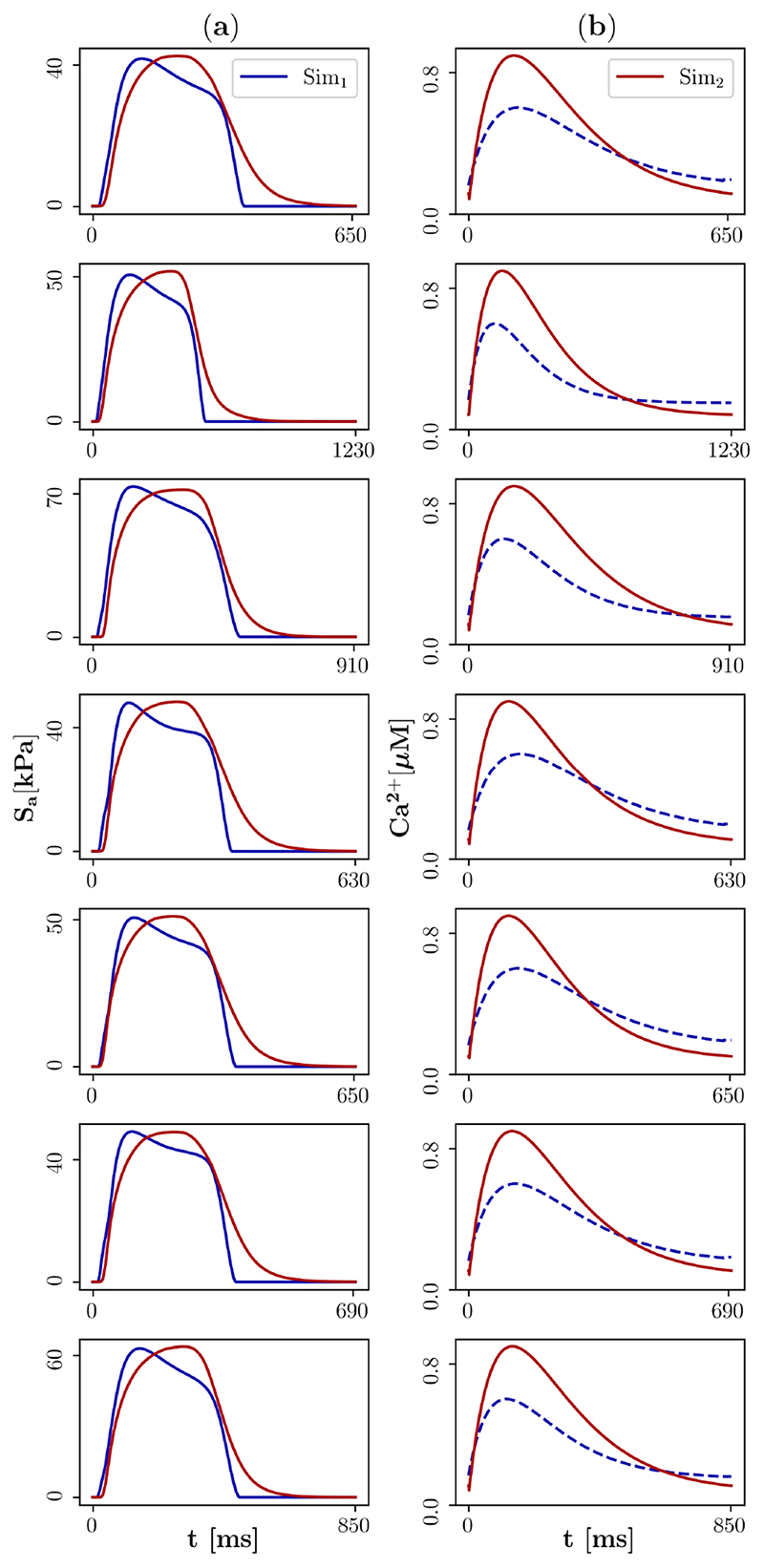
Comparison of simulated cellular active stress and intracellular calcium concentration. The blue lines represent the median nodal traces obtained from the simulations that were produced by the 3D model of human left ventricular EM in the first step (Sim_1_). The red lines represent the traces that were produced by the 0D model of cellular EM in the second step of the active mechanics personalisation approach (Sim_2_). (**a**) Cellular active stress traces (solid line represents a target for the personalisation), (**b**) intracellular calcium concentration traces (dashed line does not represent a target for the personalisation).

Median traces of nodal cellular active stress and fibre stretch obtained from the simulations of the personalised 3D models of human LV EM were then used as targets to personalise the Land model and the Rice model in the second step. The personalisation was done based on the 0D model of cellular EM and together with the stiffness parameter, a total number of ten parameters were included in the fitting. The number of iterations until convergence was between 8561 and 20,766 (Table 1). The estimated parameters are given in Table A7. Figure 5a compares the median nodal cellular active stress traces obtained in the first step with the cellular active stress traces obtained in the second step. For all cases, the maximum active stresses and transient durations at 30% decay from the maximum were in line with each other. This is a consequence of the weighting in the cost function. In contrast, the shapes in the plateau phase were slightly different and the resting value was achieved later after personalisation in the second step. Figure 5b compares the [[*Ca* ^2+^]_i_ traces. Please note that the median traces of nodal [*Ca* ^2+^]_i_ are not targets for the personalisation in the second step. For all cases, the resting value after personalisation in the second step was at the lower bound and the maximum value was at the upper bound. This allowed larger transient durations of the cellular active stress traces. The stretch traces are not shown since the only important information is whether the minimum could be matched. Minimum fibre stretches of the median nodal traces ranged from 0.82 to 0.86 and matches were found for all cases. To this end, the stiffness parameter *a*_p_ of the Land model of cellular passive stress evolution was fitted between 47.3 kPa and 86.5 kPa with a mean of 62.4 kPa (SD: 12.5 kPa).

Finally, the personalised models were incorporated in the 3D EM model to simulate one heart beat and to compare pressure and volume traces. The simulated pressure–volume loops and the individual pressure and volume traces were in good agreement with those produced by the personalised models in the first step and consequently also with the clinical data (Figure 4). The mean relative differences between the simulated and clinical values of *P*_max_ and *PTD*_90_ remained almost unchanged: 5.5% (SD: 2.3%) and 2.9% (SD: 1.5%), respectively. For ${\frac{\text{d}P}{\text{d}t}|}_{\text{max}}$ and ${\frac{\text{d}P}{\text{d}t}|}_{\text{min}}$ , the mean relative differences increased 22.5% (SD: 21.8%) and 39.9% (SD: 21.3%), respectively. The mean relative difference between the simulated and clinical *SV* also remained almost unchanged 11.1% (SD: 6.2%).

### 3.3 Robustness Analyses

#### 3.3.1 Clinical Data Uncertainty

**Figure 6:**
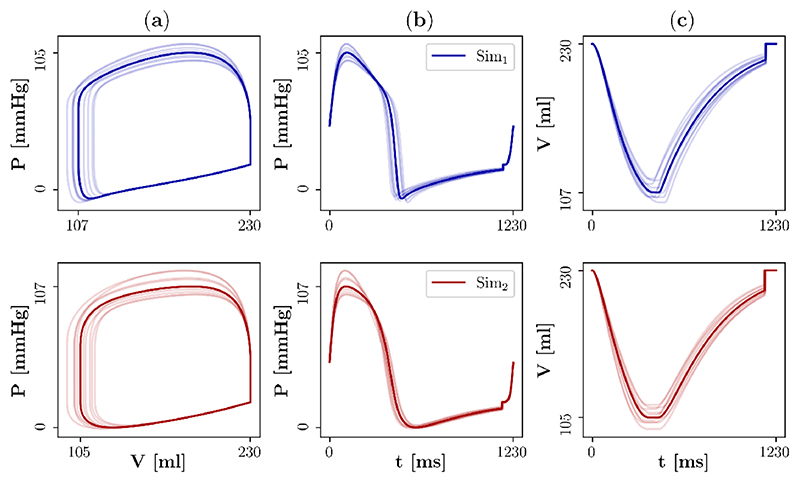
Effect of clinical data uncertainty on simulated left ventricular pressure and volume in the two steps of the active mechanics personalisation approach. The blue/red lines represent the simulation data (Sim1, Sim2) that were produced by the 3D model of human left ventricular EM in the first/second step of the active mechanics personalisation approach. The active mechanics personalisation was performed based on ten samples of clinical biomarkers (±10% around the measured value) and the resulting pressures and volumes (light colours) are compared to those that resulted from the personalisation based on the measured values (bold colours). (**a**) Pressure–volume loops, (**b**) pressure traces, (**c**) volume traces.

The robustness against clinical data uncertainty was analysed for the case 02-CoA. For this purpose, the active mechanics personalisation was performed based on ten samples of clinical biomarker values that were within a range of ±10% around the measured values. Tables 3–6 give the estimated parameter values, the resulting pressure and volume biomarker values, and the respective differences relative to the original values for the first and the second step. In the first step, the mean relative differences of the Tanh model parameter values were all below 8.0% and the mean relative differences of the VFM parameter values were similar with 10.8% (SD: 8.1%) for *R*_AVf_ and 5.0% (SD: 3.3%) for *R*_MVf_. The relative differences of the resulting pressure and volume biomarker values were all below 6.5%. In the second step, the mean relative difference for the Land parameters [Ca^2+^]_50_ref__ and *β*_1_ was comparable to the mean relative difference for the Tanh parameters: 1.3% (SD: 3.3%) and 0.3% (SD: 0.2%). For the Land model parameters *k*_UW_, *n*_TRPN_, *n*_Tm_, the mean relative difference was larger: 32.7% (SD: 40.4%), 27.0% (SD: 16.4%), 11.5% (SD: 12.4%). The mean relative differences of the Rice parameter values and the stiffness parameter *a*_p_ (results not shown) were all below 5.4%. The relative differences of the resulting pressure and volume biomarker values were below 7.0% except for ${\frac{\text{d}P}{\text{d}t}|}_{\text{max}}$ (10.5%; SD: 7.3%). The pressure–volume loops and the individual pressure and volume traces are compared in Figure 6.

#### 3.3.2 Initial Guess Variation

The robustness against variation of initial guesses was analysed for case 02-CoA. Five samples of initial guesses within a range of ±50% around the default values were used to perform the active mechanics personalisation. Tables 7–10 give the estimated parameter values, the resulting pressure and volume biomarker values, and the respective differences relative to the results produced by the default initial guesses for the first and the second step.

In the first step, the mean relative differences for the Tanh model parameters *S*_*max*_ref__, *τ*_SD_, *T*_CR_, and the VFM parameter *R*_MVf_ were below 2.9%. For *τ*_*SR*_ref__ (10.9%; SD: 11.5%) and in particular for *R*_AVf_ (30.4%; SD: 28.5%), they were larger.

In the second step, the mean relative differences for the Land model parameters *β*_1_ and [Ca^2+^]_50_ref__, for the Rice model parameters [Ca^2+^]_res_, [Ca^2+^]_max_, and for the stiffness parameter *a*_p_ (results not shown) were below 2.8%. For the remaining half of the parameter set, they were up to 59.3%. Large relative differences were mainly due to outliers in individual samples which is also indicated by the corresponding large standard deviations (up to 85.2%). The mean relative differences of the resulting pressure and volume biomarker values were below 3.4% in the first step and below 12.8% in the second step.

## 4 Discussion

This study describes a novel approach for the fully automated personalisation of biophysically detailed active mechanics models in 3D EM models. Motivated by the aim to keep the computational cost low, we suggest a two-step multi-fidelity solution based on clinical cavity pressure data. Here, the purely phenomenological Tanh model [37,38] was used as LFM and the biophysically detailed Land model [39] was used as HFM. The personalisation approach was tested for seven patient cases with previously built 3D models of human LV EM. These account for all cases in the CARDIOPROOF cohort presented by Marx et al. [18] for which invasively measured LV pressure data were recorded. Since volume traces were also available, the personalisation of the Tanh model in the first step was extended to an aortic and a mitral VFM. The personalisation of the Land model in the second step was extended to the Rice model [42] that represents [*Ca* ^2+^]_i_ evolution on a purely phenomenological basis. This was done because information on [*Ca* ^2+^]_i_ traces in vivo are missing but can have considerable influence on the parameter estimation in biophysically detailed models as demonstrated in Tøndel et al. [61].

### 4.1 Computational Cost

For the six successfully converged patient cases, only two to four 3D organ-scale simulations were required in the first step to personalise the Tanh model and the aortic and a mitral VFM (Table 1). The Land and the Rice model were personalised based on computationally much less expensive, single-core 0D cell-scale simulations. Here, we achieved a reduction of computational costs by reducing the number of considered model parameters to those that the cellular active stress trace is most sensitive to. To this end, a GSA was performed which demonstrated that the active stress is most sensitive to parameters related to the troponin C kinetics and [*Ca* ^2+^]_i_ dynamics (Figure 3). This is in line with previous observations by Tøndel et al. [61]. Overall, ten parameters were included in the fitting and the number of 0D cell-scale simulations was between 8561 and 20,766 (Table 1).

### 4.2 Goodness of Fit and Robustness

For the six successfully converged patient cases, the agreement between simulated and clinical LV pressure and volume traces was good for both the personalised Tanh model and the personalised Land model (Figure 4, Table 2). Substantial differences were only found for end-diastolic volumes; this was to be expected because the 3D model of human LV EM does not account for the atrial kick. This limitation could be addressed by coupling the 3D model of human LV or biventricular (biV) EM to a closed-loop lumped 0D model, see, e.g., [11,12], which represents the function of the remaining chambers and the circulation. However, this would require estimation of additional parameters of the closed-loop model which is beyond the scope of this work.

Mirams et al. [13] highlighted the importance of considering uncertainty in real-world data when calibrating models. In specific, observational uncertainty accounts for errors in the measurement process and residual uncertainty describes intrinsic and extrinsic variability in the biological system that is studied. We therefore tested the robustness of the presented personalisation approach against clinical data uncertainty which was estimated to be around ±10% of the measured values in line with previous work [18,20]. While variations in the estimated parameter values and resulting biomarkers are to be expected, these should ideally be not larger than the input data variation. Indeed, the mean differences relative to the original values were below 8.0% for the Tanh model parameters, below 10.8% for the VFM parameters (Table 3), and below 5.4% for the Rice model parameters and the stiffness parameter *a*_p_. Mean relative differences of all but two Land model parameters (Table 5) were below 11.5%. The maximum was 32.7%. Moreover, the consequences for the pressure and volume biomarkers were small: mean differences relative to the original values were below 6.5% in the first step (Table 4) and below 10.5% in the second step of the personalisation approach (Table 6, Figure 6). Overall, this demonstrates a high robustness against clinical data uncertainty.

In addition, the robustness against variation of initial guesses (by ±50%) was tested. It was found to be high in the first step. Except for the Tanh model parameter *τ*_*SR*_ref__ (10.9%) and the VFM parameter *R*_AVf_ (30.4%), the mean differences relative to the results produced by the default initial guesses were below 2.9% (Table 7) and the consequences for the pressure and volume biomarkers were small with mean differences below 3.8% (Table 8). The robustness was found to be lower in the second step. For two Land model parameters (*β*_1_, [*Ca* ^2+^]_50_ref__), two Rice model parameters ([*Ca* ^2+^]_res_, [*Ca* ^2+^]_max_), and the stiffness parameter *a*_p_, the mean relative differences were below 2.8%, but they were up to 59.3% for the other parameters. However, large mean relative differences resulted mainly from outliers (Table 9). The consequences for the pressure and volume biomarkers were larger with mean differences up to 12.8% (Table 10). The robustness to initial value variation of the second step may be improved by considering other variants of the differential evolution method [53].

### 4.3 Comparison to Other Active Mechanics personalisation Approaches

Kayvanpour et al. [23] presented an personalisation approach for an active mechanics model that was integrated in 3D models of human biV EM. They used a purely phenomenological model published in Sermesant et al. [62] to describe the evolution of cellular active stress. The parameter that controls the maximum was estimated together with a parameter of the passive stress based on clinical ejection fraction, stroke volume, end-diastolic and end-systolic volume, and end-diastolic and end-systolic pressure. Asner et al. [24] presented a personalisation approach for an active mechanics model that was integrated in 3D models of human LV mechanics and the cellular active stress was multiplicatively decomposed into a reference stress and a factor that accounts for stretch dependency. They treated the reference stress as parameter, which they estimated for various time points in the cardiac cycle based on clinical wall displacements and cavity pressures. In contrast to the wall displacements, the cavity pressures were not measured but derived from an empiric reference trace [63] that was adjusted based on estimates of the end-diastolic and the maximum pressure, and the time points of mitral and aortic valve opening and closing. Finsberg et al. [25] presented a personalisation approach for an active mechanics model that was integrated in 3D models of human biV mechanics. They estimated the local cellular active stress directly over time based on clinical cavity volumes and circumferential strains. Moreover, they presented an analogue for the active strain approach and estimated the local cellular active strain over time instead. Marx et al. [18] presented a personalisation approach similar to the first step of our approach. Integrated in the 3D models of human LV EM that were also used in this study, they estimated the parameters of the Tanh model [37,38] based on the corresponding clinical pressure biomarkers. However, in contrast to our approach, they set the duration of the cellular active stress transient to be the RT interval of an available clinical electrocardiogram because they were only interested in the systolic phase.

The vital difference between our approach and the described previous works is that it cannot only be used for purely phenomenological but also for biophysically detailed models. To the best of the authors knowledge, only one other approach for that purpose has been published. Longobardi et al. [27] applied BHM based on GPR models to personalise an active mechanics model that was integrated in a 3D model of rat biV EM. The evolution of cellular active stress was described by the biophysically detailed model published in Land et al. [64]. While GPR models are even cheaper to solve than 0D models, the generation of a sufficiently large training data set for highly accurate GPR models requires a high number of 3D organ-scale simulations. This limits the efficiency as the number of 3D organ-scale simulations predominantly determines the computational cost of the personalisation approach. (Considering eight parameters), Longobardi et al. [27] reported on 1024 simulations to generate training data for the GPR models being used in the first wave of BHM and at each successive wave, the GPR models were updated by training data that is generated by another 256 simulations. In comparison, only between two and four 3D organ-scale simulations were required in the presented approach.

### 4.4 Limitations

While the results of this study are very promising to further the development of cardiac digital twins, some limitations have to be considered. First, the clinical cavity pressure data used for the personalisation approach can only be collected invasively which not only entails considerable efforts but is also associated with certain risks for the patient. Nevertheless, as was done previously for the LV [24], the required data could also be derived by utilising an empiric reference pressure trace that is adjusted based on non-invasive measurements [63]. For patient-specific adjustments, [63] used the time points of aortic and mitral valve opening and closing determined by echocardiography and [24] further used estimates of the end-diastolic [65,66], and the maximum pressure [67] based on phase-contrast MRI and cuff pressure measurements, respectively. Second, the parameter constraints were selected based on two studies [39,61] or were set to ±50% of the original values. Constraining parameters in personalisation approaches is crucial and, therefore, it is desirable to extend the database in the future. Third, the approach was tested for a rather small cohort of seven patient cases and only for the active mechanics of the LV. Kayvanpour et al. [23], Finsberg et al. [25], Longobardi et al. [27] applied their personalisation approach to the active mechanics of both ventricles and an extension of our approach to multiple chambers could also be done straightforwardly. Yet, this work focuses on the methodology of the novel multi-fidelity approach and tests for larger cohorts and more chambers will be left to future studies. In this regard, machine learning techniques could be employed to handle the challenges that arise in big data [68] and, hence, to further speed up parameter calibration. Fourth, as data and results were used from a previous work by Marx et al. [18], we also applied active stresses only in fibre direction. However, several studies [69–71] have shown that active stresses in the cross-fibre direction can be as large as 40% of those in fibre directions due to a dispersion of cardiomyocytes in tissue. This was already considered in Finsberg et al. [25] and recently in a mechanistically more accurate mechanical framework in Augustin et al. [11] and we see no obstacle in using other active mechanical models for the presented approach. Fifth, the representation of cellular EP and the [*Ca* ^2+^]_i_ evolution was simplified. There are numerous biophysically detailed models of cellular EP and [*Ca* ^2+^]_i_ evolution available, e.g., [72], and if mechanistic detail is crucial for the study purpose, the simplified models have to be substituted. Finally, although we could show that the approach is widely robust to variations of initial guesses, we cannot prove uniqueness of the estimated parameters. Further, we cannot provide a rigorous proof that the fixed point approach given in Section 2.3.1 will always converge. In one case, we also encountered parameter values that caused the 3D organ-scale simulation to fail. Here, parameter constraints may improve the stability.

## 5 Conclusions

A novel approach for the fully automated personalisation of biophysically detailed active mechanics models was presented. The great strength of this approach lies in its efficiency with only a few 3D organ-scale simulations needed. Further, the application to a cohort of seven patient cases demonstrated an accurate and robust estimation of model parameters that resulted in good agreement of simulated and clinical LV pressure and volume traces. This also holds true when uncertainty in the clinical data and variations of initial guesses were taken into account. Thus, the presented workflow constitutes a further step forward towards the personalised modelling of active mechanical behaviour in the heart. As such, this approach is considered highly suitable for integration in workflows for building digital twins of cardiac EM—from a single patient to an entire cohort.

## Supplementary Material

Appendices

## Figures and Tables

**Table 1:** Mesh information, CPU times, and iteration numbers for each patient case. Given is the number of nodes and elements of the patient-specific FE mesh, the cardiac cycle length, and the times per iteration and number of iterations for personalising the low-fidelity model (LFM) in the first step (3D EM model) and the high fidelity model (HFM) in the second step (0D EM model). 3D simulations in the first step were run on ARCHER2 using 128 cores and 0D simulations in the second step were run on a desktop computer using 1 core.

Patient Case	# Nodes (-)	# Elements (-)	Cycle Length (ms)	Time/It LFM (s)	Time/It HFM (s)	# It LFM (-)	# It HFM (-)
01-CoA	159,948	806,430	659	4834.7	0.033	2	13,211
02-CoA	162,188	835,516	1231	7945.4	0.061	3	16,061
03-CoA	63,804	301,146	917	2389.4	0.045	4	17,261
04-CoA	96,176	487,132	631	2249.1	0.031	9	8561
05-CoA	126,981	652,012	654	3434.8	0.032	3	16,211
06-CoA	165,508	853,717	697	5407.9	0.036	4	14,111
07-CoA	82,212	394,690	852	2591.8	0.042	3	20,766

**Table 2:** Simulated and clinical LV pressure and volume biomarker values for each patient case. Clinical data are given in the first panel and results of the first and the second step of the active mechanics personalisation approach are given in the second and third panel. The goodness of fit is measured as relative difference between simulated and clinical value and given in brackets.

Patient Case	*P*_max_ (mmHg)	${\frac{\text{d}P}{\text{d}t}|}_{\text{max}}$ (mmHg ms^−1^)	${\frac{\text{d}P}{\text{d}t}|}_{\text{min}}$ (mmHg ms^−1^)	PTD_90_ (ms)	SV (mL)
01-CoA	119	2.7	−2.9	309	100
	115 (3.0%)	1.9 (30.9%)	−2.2 (26.7%)	330 (6.7%)	90 (10.4%)
	125 (5.1%)	2.8 (3.7%)	−1.2 (59.4%)	316 (2.1%)	92.6 (7.4%)
02-CoA	105	1.3	−1.5	452	115
	105 (<0.1%)	1.2 (5.3%)	−1.5 (2.5%)	460 (1.9%)	123 (6.8%)
	107 (1.5%)	1.5 (18.5%)	−0.9 (39.8%)	446 (1.2%)	126 (8.6%)
03-CoA	121	1.9	−1.2	450	46
	114 (5.7%)	1.7 (12.7%)	−1.3 (5.4%)	448 (0.3%)	46 (0.1%)
	112 (7.8%)	2.5 (28.7%)	−1.2 (1.2%)	431 (4.1%)	44 (4.0%)
04-CoA	129	3.6	−3.1	290	68
	126 (2.7%)	3.2 (12.0%)	−3.1 (0.9%)	290 (<0.1%)	53 (20.9%)
	126 (2.6%)	3.8 (5.7%)	−1.3 (55.8%)	302 (4.1%)	52 (23.6%)
05-CoA	133	2.7	−2.3	309	92
	120 (10.1%)	2.4 (13.8%)	−2.5 (5.7%)	309 (0.2%)	81 (11.5%)
	123 (7.4%)	3.4 (22.9%)	−1.1 (54.1%)	310 (0.5%)	83 (10.1%)
06-CoA	152	3.2	−2.7	332	100
	139 (8.7%)	2.7 (15.2%)	−2.8 (6.3%)	332 (0.1%)	87 (12.5%)
	142 (6.4%)	3.4 (6.8%)	−1.2 (54.4%)	345 (4.1%)	83 (16.5%)
07-CoA	110	1.2	−1.2	412	55
	101 (7.7%)	1.3 (5.2%)	−1.2 (6.3%)	409 (0.6%)	60 (7.6%)
	101 (7.5%)	2.1 (71.2%)	−1.0 (14.7%)	394 (4.3%)	59 (7.5%)

**Table 3:** Results of the clinical data uncertainty robustness analysis for the patient case 02-CoA. Estimated model parameter values of the first step of the active mechanics personalisation approach are given. The original values are listed in the first row and means (M), standard deviations (SD), minima (Min), and maxima (Max) of ten samples are listed in the subsequent rows. Means, standard deviations, minima, and maxima of the relative differences are given in brackets below.

	S_max_ref__ (kPa)	τ_SR_ref__ (ms)	τ_SD_ (ms)	T_CR_ (ms)	R_AVf_ (mmHg mL s^−1^)	R_MVf_ (mmHg mL s^−1^)
Original	103.2	30.1	48.8	529.3	0.0125	0.0794
Mean	103.4 (7.8%)	30.7 (8.0%)	48.3 (4.6%)	524.9 (5.0%)	0.0112 (10.8%)	0.0795 (5.0%)
SD	9.4 (4.8%)	2.6 (3.2%)	2.7 (3.2%)	30.3 (2.9%)	0.0015 (8.1%)	0.0048 (3.3%)
Min	88.9 (0.9%)	26.5 (2.8%)	43.7 (0.1%)	478.9 (0.4%)	0.0090 (0.4%)	0.0728 (<0.1%)
Max	118.8 (15.1%)	33.6 (13.9%)	51.9 (10.4%)	572.2 (9.5%)	0.0138 (23.0%)	0.0878 (10.6%)

**Table 4:** Results of the clinical data uncertainty robustness analysis for the patient case 02-CoA. LV pressure and volume biomarker values of the first step of the active mechanics personalisation approach are given. The original values are listed in the first row and means (M), standard deviations (SD), minima (Min), and maxima (Max) of ten samples are listed in the subsequent rows. Means, standard deviations, minima, and maxima of the relative differences are given in brackets below.

	*P*_max_ (mmHg)	${\frac{\text{d}P}{\text{d}t}|}_{\text{max}}$ (mmHg ms^−1^)	${\frac{\text{d}P}{\text{d}t}|}_{\text{min}}$ (mmHg ms^−1^)	PTD_90_ (ms)	SV (mL)
Original	105	1.2	−1.5	460	123
Mean	105 (3.6%)	1.2 (6.5%)	−1.5 (4.7%)	457 (5.4%)	122 (4.3%)
SD	4 (2.2%)	0.1 (3.4%)	0.1 (2.5%)	28 (2.9%)	6 (2.8%)
Min	99 (0.3%)	1.1 (1.5%)	−1.6 (0.7%)	416 (0.9%)	112 (0.2%)
Max	112 (6.5%)	1.3 (12.0%)	−1.4 (9.2%)	502 (9.6%)	132 (9.2%)

**Table 5:** Results of the clinical data uncertainty robustness analysis for the patient case 02-CoA. Estimated model parameter values of the second step of the active mechanics personalisation approach are given. The original values produced by the default initial guesses are listed in the first row and means (M), standard deviations (SD), minima (Min), and maxima (Max) of ten samples are listed in the subsequent rows. Means, standard deviations, minima, and maxima of the relative differences are given in brackets below.

	n_TRPN_ (-)	β_1_ (-)	n_Tm_ (-)	[Ca^2+^]_50_ref__ (μM)	k_UW_ (ms^−1^)	[Ca^2+^]_res_ (μM)	[Ca^2+^]_max_ (μM)	τ_CR_ (ms)	τ_CD_ (ms)
Original	3.22	−1.21	5.47	0.501	0.046	0.076	0.900	164.4	149.0
Mean	3.60 (27.0%)	−1.20 (0.3%)	4.94 (11.5%)	0.507 (1.3%)	0.055 (32.7%)	0.075 (1.6%)	0.900 (<0.1%)	158.1 (5.4%)	154.0 (4.8%)
SD	0.94 (16.4%)	<0.01 (0.2%)	0.76 (12.4%)	0.017 (3.3%)	0.022 (40.4%)	<0.001 (0.4%)	<0.001 (<0.1%)	7.0 (2.1%)	6.2 (2.4%)
Min	2.42 (9.1%)	−1.21 (0.0%)	3.01 (1.8%)	0.500 (0.1%)	0.036 (1.1%)	0.075 (0.8%)	0.899 (<0.1%)	147.7 (2.0%)	141.7 (0.3%)
Max	5.00 (55.1%)	−1.20 (0.5%)	5.79 (44.9%)	0.557 (11.2%)	0.109 (138.8%)	0.076 (2.0%)	0.900 (0.1%)	173.5 (10.2%)	162.2 (8.8%)

**Table 6:** Results of the clinical data uncertainty robustness analysis for the patient case 02-CoA. LV pressure and volume biomarker values of the second step of the active mechanics personalisation approach are given. The original values produced by the default initial guesses are listed in the first row and means (M), standard deviations (SD), minima (Min), and maxima (Max) of ten samples are listed in the subsequent rows. Means, standard deviations, minima, and maxima of the relative differences are given in brackets below.

	P_max_ (mmHg)	${\frac{\text{d}P}{\text{d}t}|}_{\text{max}}$ (mmHg ms^−1^)	${\frac{\text{d}P}{\text{d}t}|}_{\text{min}}$ (mmHg ms^−1^)	PTD_90_ (ms)	**SV** (mL)
Original	107	1.5	−0.9	446	126
Mean	108 (5.2%)	1.6 (10.5%)	−1.0 (7.0%)	442 (4.0%)	124 (4.3%)
SD	6 (3.6%)	0.2 (7.3%)	0.1 (4.6%)	20 (2.3%)	6 (2.7%)
Min	100 (0.2%)	1.3 (0.5%)	−1.1 (1.2%)	412 (0.2%)	115 (0.2%)
Max	119 (11.3%)	1.8 (23.2%)	−0.8 (16.8%)	473 (7.6%)	135 (8.6%)

**Table 7:** Results of the initial guess variation robustness analysis for the patient case 02-CoA. Estimated model parameters of the first step of the active mechanics personalisation approach are given. The original values produced by the default initial guesses are listed in the first row and means (M), standard deviations (SD), minima (Min), and maxima (Max) of five samples are listed in the subsequent rows. Means, standard deviations, minima, and maxima of the relative differences are given in brackets below.

	S_max_ref__ [kPa]	τ_SR_ref__ [ms]	τ_SD_ [ms]	T_CR_ [ms]	R_AVf_ [mmHg mL s^−1^]	R_MVf_ [mmHg mL s^−1^]
Original	103.2	30.1	48.8	529.3	0.0125	0.0794
Mean	102.3 (1.2%)	27.6 (10.9%)	48.0 (2.0%)	517.6 (2.2%)	0.0142 (30.4%)	0.0817 (2.9%)
SD	1.4 (1.1%)	3.7 (11.5%)	0.9 (1.5%)	2.3 (0.4%)	0.0036 (28.5%)	0.0001 (0.1%)
Min	100.4 (0.2%)	20.7 (1.1%)	46.9 (0.1%)	514.1 (1.7%)	0.0101 (0.5%)	0.0815 (2.7%)
Max	104.0 (2.7%)	31.2 (33.0%)	49.2 (3.9%)	520.2 (2.9%)	0.0192 (70.6%)	0.0817 (3.0%)

**Table 8:** Results of the initial guess variation robustness analysis for the patient case 02-CoA. Pressure and volume biomarker values of the first step of the active mechanics personalisation approach are given. The original values produced by the default initial guesses are listed in the first row and means (M), standard deviations (SD), minima (Min), and maxima (Max) of five samples are listed in the subsequent rows. Means, standard deviations, minima, and maxima of the relative differences are given in brackets below.

	P_max_ [mmHg]	${\frac{\text{d}P}{\text{d}t}|}_{\text{max}}$ [mmHg ms^−1^]	${\frac{\text{d}P}{\text{d}t}|}_{\text{min}}$ [mmHg ms^−1^]	PTD_90_ [ms]	SV [mL]
Original	105	1.2	−1.5	460	123
Mean	106 (1.0%)	1.2 (3.4%)	−1.5 (3.1%)	451 (1.9%)	122 (1.4%)
SD	106 (0.5%)	<0.1 (3.6%)	<0.1 (2.6%)	1 (0.3%)	1 (0.6%)
Min	104 (0.3%)	1.2 (0.5%)	−1.6 (0.9%)	449 (1.5%)	121 (0.3%)
Max	107 (1.6%)	1.3 (10.4%)	−1.5 (8.1%)	453 (2.4%)	123 (1.9%)

**Table 9:** Results of the initial guess variation robustness analysis for the patient case 02-CoA. Estimated model parameters of the second step of the active mechanics personalisation approach are given. The original values produced by the default initial guesses are listed in the first row and means (M), standard deviations (SD), minima (Min), and maxima (Max) of five samples are listed in the subsequent rows. Means, standard deviations, minima, and maxima of the relative differences are given in brackets below.

	n_TRPN_ []	β_1_ []	n_T_m__ []	[Ca^2+^]_50_ref__ [μM]	k_UW_ [ms^−1^]	[Ca^2+^]_res_ [μM]	[Ca^2+^]_max_ [μM]	τ_CR_ [ms]	τ_CD_ [ms]
Original	3.22	−1.21	5.47	0.501	0.046	0.076	0.900	164.4	149.0
Mean	3.55 (33.6%)	−1.21 (0.5%)	4.35 (23.6%)	0.512 (2.3%)	0.071 (59.3%)	0.075 (0.1%)	0.900 (2.8%)	142.4 (28.3%)	151.5 (10.4%)
SD	1.21 (19.4%)	<0.01 (0.3%)	1.13 (17.2%)	0.014 (2.8%)	0.004 (85.2%)	<0.001 (0.6%)	<0.001 (0.1%)	52.3 (19.7%)	18.8 (7.4%)
Min	2.32 (2.9%)	−1.22 (0.2%)	3.00 (4.7%)	0.500 (<0.1%)	0.042 (1.1%)	0.075 (0.2%)	0.897 (<0.1%)	77.2 (6.9%)	123.3 (1.3%)
Max	4.99 (54.8%)	−1.20 (1.1%)	5.90 (45.1%)	0.538 (7.4%)	0.149 (226.9%)	0.077 (2.0%)	0.900 (0.3%)	192.4 (53.1%)	178.8 (20.0%)

**Table 10:** Results of the initial guess variation robustness analysis for the patient case 02-CoA. Pressure and volume biomarker values of the second step of the active mechanics personalisation approach are given. The original values produced by the default initial guesses are listed in the first row and means (M), standard deviations (SD), minima (Min), and maxima (Max) of five samples are listed in the subsequent rows. Means, standard deviations, minima, and maxima of the relative differences are given in brackets below.

	*P*_max_ [mmHg]	${\frac{\text{d}P}{\text{d}t}|}_{\text{max}}$ [mmHg ms^−1^]	${\frac{\text{d}P}{\text{d}t}|}_{\text{min}}$ [mmHg ms^−1^]	PTD_90_ [ms]	SV [mL]
Original	107	1.5	−0.9	446	126
Mean	112 (6.6%)	1.7 (12.8%)	−0.9 (5.5%)	422 (9.7%)	120 (10.7%)
SD	9 (7.4%)	0.3 (15.6%)	0.1 (3.3%)	47 (6.6%)	15 (6.5%)
Min	105 (0.4%)	1.4 (0.2%)	−1.0 (0.8%)	351 (3.6%)	100 (1.5%)
Max	127 (19.4%)	2.1 (41.6%)	−0.8 (9.5%)	473 (21.3%)	137 (20.6%)

## Abbreviations

The following abbreviations are used in this manuscript:

3DWH: three-dimensional-whole-heart
AR: aortic root
BHM: Bayesian history matching
biV: biventricular
CoA: aortic coarctation
CEM: intracellular calcium concentration evolution model
EM: electromechanics
EP: electrophysiology
FE: finite element
GSA: global sensitivity analysis
GPR: Gaussian process regression
HFM: high-fidelity model
LFM: low-fidelity model
LV: left ventricle/ventricular
MRI: magnet resonance imaging
SD: standard deviation
VFM: valve flow model
