# Supplementary material for: An Integrated Workflow for Building Digital Twins of Cardiac Electromechanics—A Multi-Fidelity Approach for Personalising Active Mechanics
Source: Mathematics (Basel). Author manuscript; Available in PMC 2022 Mar 15. (PMC7612499; doi:10.3390/math10050823)
Supplement: Appendices [file EMS143818-supplement-Appendices.pdf]

Appendix A. Models of Cellular Mechanics and the Intracellular Calcium Concentration Evolution

Appendix A.1. Tanh Model of Cellular Active Stress Evolution

The Tanh model [37,38] describes the evolution of cellular active stress as function of fibre stretch and the electrical activation time:

$$S_{\mathrm{a}}(t_{\mathrm{a}},\lambda)=\begin{cases}0 & \text{if } t\leq t_{\mathrm{s}}(t_{\mathrm{a}},t_{\mathrm{emd}})\\ S_{max_{\mathrm{ref}}}\phi\tanh^2\left(\frac{t}{\tau_{\mathrm{SR}}}\right)\tanh^2\left(\frac{t_{\mathrm{CR}}-t}{\tau_{\mathrm{SD}}}\right) & \text{if } t_{\mathrm{s}}(t_{\mathrm{a}},t_{\mathrm{emd}})<t\leq t_{\mathrm{e}}\\ 0 & \text{if } t\geq t_{\mathrm{e}}.\end{cases} \tag{A1}$$

Here, $S_{max_{\text{ref}}}$ is the maximum isometric cellular active stress and

$$\phi = \tan\big(a_6(\lambda - a_7)\big) \tag{A2}$$

is a nonlinear function that describes stretch effects on the generated active stress. The coefficient a_6 corresponds to the degree of the stretch dependency and the coefficient a_7 is the fibre stretch below which no active stress can be generated. Furthermore,

$$\tau_{\text{SR}} = \tau_{\text{SR}_{\text{ref}}} + a_4(1 - \phi). \tag{A3}$$

is the time constant of the contraction phase (rise time constant) that accounts for stretch effects. The isometric value is $\tau_{\text{SR}_{\text{ref}}}$ and the coefficient a_4 corresponds to the degree of the stretch dependency. The time constant of the relaxation phase (decay time constant) is denoted by τ_{SR} and t_{CR} is the duration of the entire contraction–relaxation cycle (transient). The contraction–relaxation cycle starts at t_{s} and ends at t_{e}. The starting time is the electrical activation time t_{a} plus some electromechanical delay t_{emd} to account for the time lag between electrical activation and the onset of contraction. The parameter values are given in Table A1.

Table A1. Parameters of the Tanh model [37,38].

Parameter	Unit	Value
$S_{a_{\text{ref}}}$	(kPa)	100
τ_{SR}	(ms)	40
τ_{SD}	(ms)	110
t_{CR}	(ms)	550
a_4	(ms)	500
a_6	(-)	5
a_7	(-)	0.7
t_{emd}	(ms)	15

Appendix A.2. Land Model of Cellular Active Stress Evolution

The Land model [39] describes the evolutionn of cellular active stress as function of $[Ca^{2+}]_{\text{i}}$ and both the fibre stretch λ and the fibre stretch rate $\frac{\text{d}\lambda}{\text{d}t}$. It is composed of a model of thin filament kinetics and a model of the cross bridge cycle. The model of thin filament kinetics describes the interactions of Ca^{2+}, troponin C, troponin I, and tropomyosin that control the availability of myosin binding sites on actin. The dynamics of the interaction between Ca^{2+} and troponin C is described by

$$\frac{\text{d}CaTRPN}{\text{d}t} = k_{\text{TRPN}}\left(\left(\frac{[Ca^{2+}]_{\text{i}}}{[Ca^{2+}]_{50}(\lambda)}\right)^{n_{\text{TRPN}}}(1 - CaTRPN) - CaTRPN\right), \tag{A4}$$

where $CaTRPN$ is the fraction of regulatory troponin C sites with bound Ca^{2+}, k_{TRPN} represents the unbinding rate of Ca^{2+} from troponin C, and n_{TRPN} is the cooperativity of the binding between Ca^{2+} and troponin C. The value of $[Ca^{2+}]_{\text{i}}$ at which half of the maximum active stress generated is denoted by $[Ca^{2+}]_{50}$. Stretch effects are phenomenologically captured by

$$[Ca^{2+}]_{50} = [Ca^{2+}]_{50_{\text{ref}}} + \beta_1(\min(\lambda, 1.2) - 1), \tag{A5}$$

where $[Ca^{2+}]_{50_{\text{ref}}}$ is the isometric value that is scaled by β_1. The fraction of regulatory troponin C sites with bound Ca^{2+} drives the unblocking of tropomyosin:

$$\frac{\text{d}B}{\text{d}t} = k_{\text{B}}CaTRPN^{\frac{-n_{\text{Tm}}}{2}}U - k_{\text{U}}CaTRPN^{\frac{n_{\text{Tm}}}{2}}B, \tag{A6}$$

where B is the fraction of blocked myosin binding sites on actin,

$$k_{\mathrm{B}} = \frac{k_{\mathrm{U}} CaTRPN^{n_{\mathrm{Tm}}}}{1 - r_{\mathrm{S}} - (1 - r_{\mathrm{S}}) r_{\mathrm{W}}} \tag{A7}$$

and k_U are the troponin I and tropomyosin rate constants, respectively, n_{Tm} is the steady-state relation between $CaTRPN$ and the fraction of unblocked binding sites $(1 - B)$, and U is the fraction of the unblocked myosin binding sites with no cross bridges formed.

The model of the cross bridge cycle accounts for three states: the unbound, the weak (pre-powerstroke), and the strong (post-powerstroke) state. It reads

$$\begin{aligned} U &= (1 - B) - S - W, \\ \frac{\mathrm{d}W}{\mathrm{d}t} &= k_{\mathrm{UW}} U - k_{\mathrm{WU}} W - k_{\mathrm{WS}} W - \gamma_{\mathrm{WU}} W, \\ \frac{\mathrm{d}S}{\mathrm{d}t} &= k_{\mathrm{WS}} W - k_{\mathrm{SU}} S - \gamma_{\mathrm{SU}} S, \end{aligned} \tag{A8}$$

where W and S are the weak, and the strong states, respectively, and k_{UW}, k_{WU}, k_{WS}, k_{SU} and are transition rates. Latter are defined by

$$\begin{aligned} k_{\mathrm{WS}} &= k_{\mathrm{UW}} \frac{1}{r_{\mathrm{W}} - 1} - k_{\mathrm{WS}}, \\ k_{\mathrm{SU}} &= k_{\mathrm{WS}} r_{\mathrm{W}} \frac{1}{r_{\mathrm{S}} - 1}, \end{aligned} \tag{A9}$$

with the steady-state ratios

$$\begin{aligned} r_{\mathrm{W}} &= \text{steady-state} \frac{W}{U + W}, \\ r_{\mathrm{S}} &= \text{steady-state} \frac{S}{U + W + S}. \end{aligned} \tag{A10}$$

The distortion-depending unbinding rates of the cross bridges are

$$\begin{aligned} \gamma_{\mathrm{WU}} &= \gamma_{\mathrm{W}} |\xi_{\mathrm{W}}|, \\ \gamma_{\mathrm{SU}} &= \begin{cases} \gamma_{\mathrm{S}}(-\xi_{\mathrm{S}} - 1) & \text{if } \xi_{\mathrm{S}} + 1 < 0 \\ \gamma_{\mathrm{S}} \xi_{\mathrm{S}} & \text{if } \xi_{\mathrm{S}} + 1 > 1 \\ 0 & \text{otherwise,} \end{cases} \end{aligned} \tag{A11}$$

and these are coupled to a distortion-decay model given by

$$\begin{aligned} \frac{\mathrm{d}\xi_{\mathrm{W}}}{\mathrm{d}t} &= A_{\mathrm{W}} \frac{\mathrm{d}\lambda}{\mathrm{d}t} - c_{\mathrm{W}} \xi_{\mathrm{W}}, \\ \frac{\mathrm{d}\xi_{\mathrm{S}}}{\mathrm{d}t} &= A_{\mathrm{S}} \frac{\mathrm{d}\lambda}{\mathrm{d}t} - c_{\mathrm{S}} \xi_{\mathrm{S}}. \end{aligned} \tag{A12}$$

Here, ξ_{W} and ξ_{S} are the stretch rate-dependent mean distortions and

$$A_{\mathrm{W}} = A_{\mathrm{S}} = \frac{A_{\mathrm{eff}} r_{\mathrm{S}}}{(1 - r_{\mathrm{S}}) r_{\mathrm{W}} + r_{\mathrm{S}}} \tag{A13}$$

are related to the magnitude of the instantaneous response to the distortion with some scaling A_{eff}, whereas

$$\begin{aligned} c_{\mathrm{W}} &= \Phi k_{\mathrm{UW}} \frac{U}{W}, \\ c_{\mathrm{S}} &= \Phi k_{\mathrm{WS}} \frac{W}{S} \end{aligned} \tag{A14}$$

are related to the magnitude of the decay rate of the distortion. The introduction of Φ eliminates a parameter by reducing two parameters $(c_\mathrm{W}, c_\mathrm{S})$ to one.

Finally, the active stress is given by

$$S_\mathrm{a}([Ca^{2+}]_\mathrm{i}, \lambda, \frac{\mathrm{d}\lambda}{\mathrm{d}t}) = h(\lambda)\frac{S_{max_\mathrm{ref}}}{r_s}[(\xi_S(\frac{\mathrm{d}\lambda}{dt}) + 1)S + \xi_\mathrm{W}(\frac{\mathrm{d}\lambda}{\mathrm{d}t})\mathrm{W}], \tag{A15}$$

where S_{max_ref} is the maximum isometric active stress and fibre stretch effects on the generated active stress are phenomenologically captured by

$$h = 1 + \beta_0(\lambda + \min(\lambda, 0.87) - 1.87), \tag{A16}$$

where β_0 represents the change in maximum active stress based on changes in filament overlap. The parameter values are given in Table A2.

Table A2. Parameters of the Land model [39] (active).

Parameter	Unit	Value
k_TRPN	(ms^{-1})	0.1
n_TRPN	(-)	2
$[Ca^{2+}]_{50_\mathrm{ref}}$	(μM)	0.805
k_U	(ms^{-1})	1
n_Tm	(-)	5
$TRPN_{50}$	()	0.35
k_UW	(ms^{-1})	0.182
k_WS	(ms^{-1})	0.012
r_W	(-)	0.5
r_S	(-)	0.25
γ_S	(-)	0.0085
γ_W	(-)	0.615
Φ	(-)	2.23
A_eff	(-)	25
β_0	(-)	2.3
β_1	(-)	−2.4
S_{max_ref}	(kPa)	120

Appendix A.3. Land Model of Cellular Passive Stress Evolution

The Land model [39] describes the evolution of cellular passive stress by a three-element model similar to a standard linear solid. It consists of an elastic spring (E1) in parallel to another elastic spring (E2) in series with a viscous dashpot (V) and reads

$$S_\mathrm{p}(\lambda) = S_{E1} + S_{E2} = S_{E1} + S_\mathrm{V}, \tag{A17}$$

with the stress components

$$S_{E1} = a_\mathrm{p}(e^{\mathrm{b_p C}} - 1), S_{E2} = a_\mathrm{p}k_\mathrm{p}C_\mathrm{S}, \tag{A18}$$

$$S_\mathrm{V} = \begin{cases} a_\mathrm{p}\eta_1\frac{\mathrm{d}C_\mathrm{V}}{\mathrm{d}t} & \text{if } \frac{\mathrm{d}C_\mathrm{V}}{\mathrm{d}t} > 0 \\ a_\mathrm{p}\eta_\mathrm{S}\frac{\mathrm{d}C_\mathrm{V}}{\mathrm{d}t} & \text{if } \frac{\mathrm{d}C_\mathrm{V}}{\mathrm{d}t} < 0' \end{cases} \tag{A19}$$

the series strain constraint

$$C = C_\mathrm{S} + C_\mathrm{V}, \tag{A20}$$

and the series stress constraint

$$S_{E2} = S_\mathrm{V}. \tag{A21}$$

Strain C is defined as $C = \lambda - 1$ with stretch defined as the ratio of the current and initial cardiomyocyte length. The stiffness parameter a_p is included in all stress components, such that it is suitable for scaling the passive stress. The parameter values are given in Table A3.

Table A3. Parameters of the Land model [39] (passive).

Parameter	Unit	Value
a_p	(kPa)	2.1
b_p	(-)	9.1
k_p	(-)	7
η_l	(ms^{-1})	200
η_S	(ms^{-1})	20

Appendix A.4. Rice Model of the Intracellular Calcium Concentration Evolution

The Rice model [42] describes the evolution of $[Ca^{2+}]_i$ as a function of the electrical activation time by

$$[Ca^{2+}]_i(t_a) = \begin{cases} [Ca^{2+}]_{res} & \text{if } t \leq t_a \\ \frac{[Ca^{2+}]_{max} - [Ca^{2+}]_{res}}{\beta} \exp\left(-\frac{t-t_s}{\tau_{CR}}\right) \exp\left(-\frac{t-t_s}{\tau_{CD}}\right) + [Ca^{2+}]_{res} & \text{if } t > t_a, \end{cases} \tag{A22}$$

with

$$\beta = \left(\frac{\tau_{CR}}{\tau_{CD}}\right)^{\frac{-1}{\left(\frac{\tau_{CR}}{\tau_{CD}} - 1\right)}} - \left(\frac{\tau_{CR}}{\tau_{CD}}\right)^{\frac{-1}{\left(1 - \frac{\tau_{CR}}{\tau_{CD}}\right)}}, \tag{A23}$$

where $[Ca^{2+}]_{res}$ and $[Ca^{2+}]_{max}$ are the resting and the maximum $[Ca^{2+}]_i$, respectively, and τ_{CR} and τ_{CR} are the time constants during the rise and the decay phase of the $[Ca^{2+}]_i$ transient, respectively. Starting time of the transient is the electrical activation time: $t_s = t_a$. The model was calibrated based on an experimentally measured $[Ca^{2+}]_i$ trace in human cardiomyocytes [43]. To this end, the unconstrained minimisation problem

$$\min_{p^{Rice}} \sum_{j=1}^{n} \left([Ca^{2+}]^{exp,j} - [Ca^{2+}]^{sim,j}(p^{Rice}) \right)^2, \tag{A24}$$

was solved to estimate the parameters p^{Rice}. Here, $j = 1, \ldots, n$ are all data points of the trace. Powell's method implemented in the library *lmfit: Non-linear least-squares minimisation and curve-fitting Python library* [56] was used. The estimated parameter values are given in Table A4.

Table A4. Parameters of the Rice model [42].

Parameter	Unit	Value
$[Ca^{2+}]_{res}$	(µM)	0.15
$[Ca^{2+}]_{max}$	(µM)	0.6
τ_{CR}	(ms)	129
τ_{CD}	(ms)	128

Appendix B. Parameter Bounds

Physiological lower and upper bounds were applied to the parameters of the Land model of cellular active and passive stress evolution and the Rice model. The bounds for the parameters of the Land model of cellular active stress evolution were either set according to Tøndel et al. [61] or according to Land et al. [39] or they were set to ±50% of the original value if no information were available. The bounds for the Rice model

parameters were set to ±50% of the original value in agreement with the $[Ca^{2+}]_i$ traces of the experimentally calibrated model cohort shown in Passini et al. [52]. The range of the stiffness factor in the Land model of cellular passive stress evolution was set to be very wide as this parameter has no physiological meaning in the context of the second step of the active mechanics personalisation approach. All bounds are given in Table A5.

Table A5. Parameter bounds used in this study. Lower bounds are denoted by LB and upper bounds are denoted by UB. If applicable, the source is given.

Parameter	Unit	LB	UB	Source
Land model (active)				
k_{TRPN}	(ms^{-1})	0.05	0.4	[61]
n_{TRPN}	(-)	1	5	[61]
$[Ca^{2+}]_{50_{\mathrm{ref}}}$	(μM)	0.5	2.0	[61]
k_{u}	(ms^{-1})	0.01	2.00	[39]
n_{Tm}	(-)	3	7	[39]
$TRPN_{50}$	(-)	0.3	0.5	[61]
k_{UW}	(ms^{-1})	0.026	0.312	[39]
k_{WS}	(ms^{-1})	0.004	0.048	[39]
r_{W}	(-)	0.25	0.75	[39]
r_{S}	(-)	0.10	0.25	[39]
γ_{S}	(-)	0.005	0.020	[39]
γ_{W}	(-)	0.05	3.00	[39]
Φ	(-)	0.1	4.0	[39]
A_{eff}	(-)	12.5	37.5	
β_0	(-)	1.15	4.60	
β_1	(-)	−3.6	−1.2	
$S_{max_{\mathrm{ref}}}$	(kPa)	90	140	[61]
Land model (passive)				
a_{p}	(kPa)	1	100	
Rice model				
$[Ca^{2+}]_{\mathrm{res}}$	(μM)	0.075	0.225	
$[Ca^{2+}]_{\mathrm{max}}$	(μM)	0.3	0.9	
τ_{CR}	(ms)	64.5	192.5	
τ_{CD}	(ms)	64	192	

Appendix C. Biomarker Definitions

The definitions of the biomarkers used in this study are illustrated in Figure A1. Based on the cavity pressure trace, the pressure biomarkers are defined as:

$$\begin{aligned} P_{\max} &= \max P(t), \\ \frac{\mathrm{d}P}{\mathrm{d}t}\Big|_{\max} &= \max_{t<t_2} \frac{\mathrm{d}P}{\mathrm{d}t}, \\ \frac{\mathrm{d}P}{\mathrm{d}t}\Big|_{\min} &= \min_{t>t_2} \frac{\mathrm{d}P}{\mathrm{d}t}, \\ PTD_{90} &= t_3 - t_1. \end{aligned} \tag{A25}$$

Here, $P_{\max}$ is the maximum, $\frac{\mathrm{d}P}{\mathrm{d}t}|_{\max}$ and $\frac{\mathrm{d}P}{\mathrm{d}t}|_{\min}$ are the maximum and minimum rates, and PTD_{90} is the transient duration at 90% decay from the maximum, measured between the time points at 10% of the amplitude.

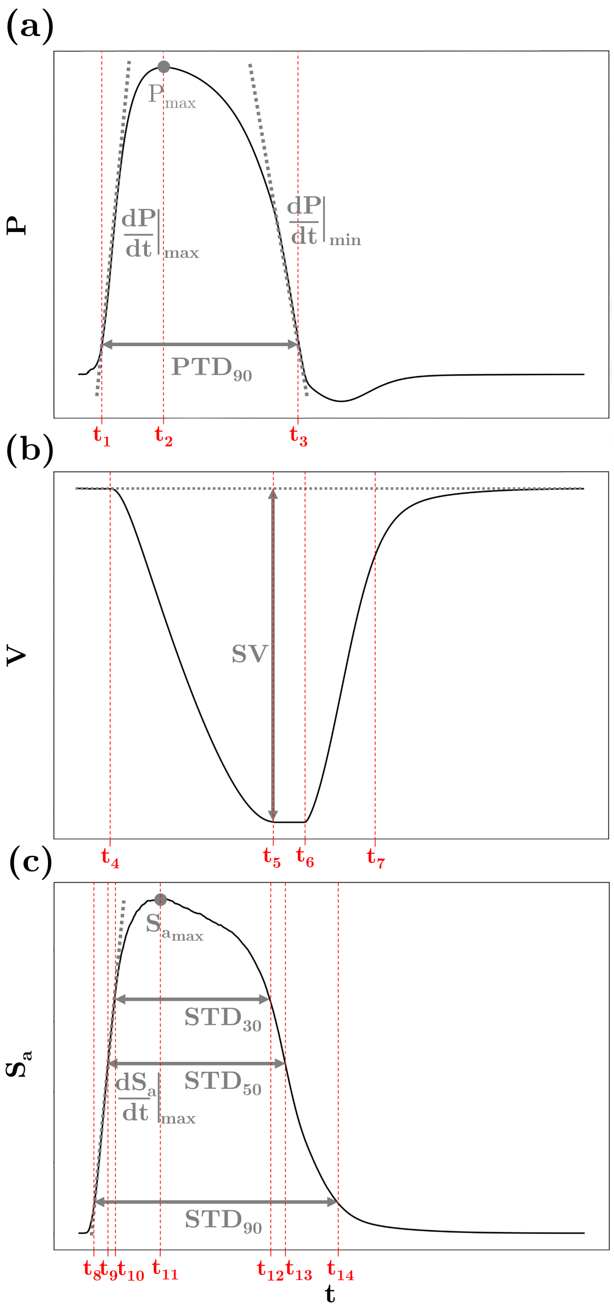

Figure A1. Biomarker definition and critical time points. (**a**) Pressure biomarkers, (**b**) volume biomarkers, and (**c**) active stress biomarkers.

Based on the cavity volume trace, the volume biomarkers are defined as:

$$
\begin{aligned}
SV &= \max V(t) - \min V(t), \\
\tau_{\mathrm{VD}} &= -\frac{1}{s_{\mathrm{VD}}}, \\
\tau_{\mathrm{VR}} &= -\frac{1}{s_{\mathrm{VR}}}.
\end{aligned} \tag{A26}
$$

Here, SV is the stroke volume, τ_{VD} is the decay time constant during the ejection phase, and τ_{VR} is the rise time constants for the filling phase until the beginning of the

atrial kick, which is defined to be the time point at 80% volume recovery, in line with [73]. The slope s_{VD} is computed by an ordinary linear regression of

$$\ln\left(\frac{V(t) - \min V(t)}{SV}\right) \tag{A27}$$

against t from t_4 to t_5 and the slope s_{VR} is computed by an ordinary linear regression of

$$\ln\left(1 - \frac{V(t) - \min V(t)}{V(t_7) - \min V(t)}\right) \tag{A28}$$

against t from t_6 to t_7.

Based on the cellular active stress trace, the active stress biomarkers are defined as:

$$\begin{aligned} S_{a_{\max}} &= \max S_{\mathrm{a}}(t), \\ \frac{dS_{\mathrm{a}}}{dt}|_{\max} &= \max_{t<t_{10}} \frac{dS_{\mathrm{a}}}{dt}, \\ STD_{30} &= t_8 - t_{12}, \\ STD_{50} &= t_9 - t_{13}, \\ STD_{90} &= t_{10} - t_{14}. \end{aligned} \tag{A29}$$

Here, $S_{a_{\max}}$ is the maximum, $\frac{dS_{\mathrm{a}}}{dt}|_{\max}$ is the maximum rate, and STD_{30}, STD_{50} and STD_{90} are the transient durations at 30%, 50%, and 90% decay from the maximum, measured between the time points at 70%, 50%, and 10% of the amplitude.

Appendix D. Penalty Formulation of the Constrained Minimisation Problem in the Second Step of the Active Mechanics Personalisation Approach

The constrained minimisation problem Equation (19) was solved as a series of unconstrained problems by application of the penalty method:

$$\begin{aligned} \min_{p_{\mathrm{HFM}}, a_{\mathrm{p}}, p_{\mathrm{CEM}}} \quad & \left[\sum_{j=1}^{n_{\mathrm{HFM}}} w^{j}_{S_{\mathrm{a}}} \left(\frac{B^{\mathrm{tar},j}_{S^*_{\mathrm{a}}} - B^{\mathrm{sim},j}_{S_{\mathrm{a}}}(p_{\mathrm{HFM}}, a_{\mathrm{p}}, p_{\mathrm{CEM}})}{B^{\mathrm{tar},j}_{S^*_{\mathrm{a}}}} \right)^2 + \right. \\ & \left. w_{\lambda} \left(\frac{B^{\mathrm{tar}}_{\lambda^*} - B^{\mathrm{sim}}_{\lambda}(p_{\mathrm{HFM}}, a_{\mathrm{p}}, p_{\mathrm{CEM}})}{B^{\mathrm{tar}}_{\lambda^*}} \right)^2 \right] + \mathrm{pen}. \end{aligned} \tag{A30}$$

where the penalty term reads as follows:

$$\begin{aligned} \mathrm{pen} =& S^2_{\mathrm{a_{res}}} + \\ & \int_0^T \left[\min(0, S_{\mathrm{a}}(t))\right]^2 \mathrm{d}t + \\ & \int_{t_{S_{\mathrm{a}_{\max}}}}^T \left[\max\left(0, \frac{\mathrm{d}S_{\mathrm{a}}(t)}{\mathrm{d}t}\right)\right]^2 \mathrm{d}t + \\ & [\min(0, CTD_{50} - 120)]^2 + \max(0, CTD_{50} - 120)]^2 + \\ & [\min(0, CTD_{90} - 220)]^2 + \max(0, CTD_{90} - 785)]^2. \end{aligned} \tag{A31}$$

Since the differential evolution method was used for solving, the parameter constraints were enforced by limiting the populations of candidate solutions to the admissible ranges.

Appendix E. Estimated Parameter Values of the Personalised Models

The values of the Tanh model parameters and the values of the VFM parameters that were estimated in the first step are given in Table A6. The values of the Land model

parameters and the Rice model parameters that were estimated in the second step are given in Table A7.

Table A6. Estimated parameter values of the personalised Tanh model and the personalised valve flow models for each patient case.

Patient Case	$S_{max_{ref}}$ (kPa)	$\tau_{SR_{ref}}$ (ms)	τ_{SD} (ms)	T_{CR} (ms)	R_{AVf} (mmHg mL s^{-1})	R_{MVf} (mmHg mL s^{-1})
01-CoA	108.4	20.5	36.5	376.4	0.0068	0.0444
02-CoA	103.2	30.1	48.8	529.3	0.0125	0.0794
03-CoA	182.3	7.0	77.5	514.8	0.0028	0.0562
04-CoA	220.4	0.89	35.0	330.0	0.0001	0.0646
05-CoA	145.2	12.5	39.2	352.4	0.0040	0.0472
06-CoA	172.1	6.1	40.3	376.0	0.0026	0.0324
07-CoA	146.8	26.1	64.9	475.5	0.0069	0.0593

Table A7. Estimated parameter values of the personalised Land model and the personalised Rice model for each patient case.

Patient Case	n_{TRPN} (-)	β_1 (-)	n_{Tm} (-)	$[Ca^{2+}]_{50_{ref}}$ (μM)	k_{UW} (ms^{-1})	$[Ca^{2+}]_{res}$ (μM)	$[Ca^{2+}]_{max}$ (μM)	τ_{CR} (ms)	τ_{CD} (ms)
01-CoA	3.88	−1.20	3.01	0.615	0.147	0.076	0.895	105.6	120.0
02-CoA	3.22	−1.21	5.47	0.501	0.046	0.076	0.900	164.4	149.0
03-CoA	4.99	−1.20	6.02	0.500	0.250	0.075	0.900	162.9	152.8
04-CoA	5.00	−1.20	5.68	0.500	0.031	0.075	0.899	140.4	67.6
05-CoA	4.82	−1.24	3.00	0.505	0.031	0.082	0.898	103.6	93.4
06-CoA	4.99	−1.20	3.48	0.511	0.029	0.076	0.899	113.8	114.5
07-CoA	4.98	−1.20	4.12	0.500	0.097	0.076	0.899	139.4	142.6

Appendix F. Convergence Behaviour for Patient Case 04-CoA in the First Step

Figure A2 illustrates the convergence behaviour in the first step of the personalisation approach for patient case 04-CoA. It shows that parameter values and cost both converge, however, after the ninth iteration, the required convergence threshold is still not reached. Ultimately, the updated set of parameter values after the ninth iteration caused the simulation to fail.

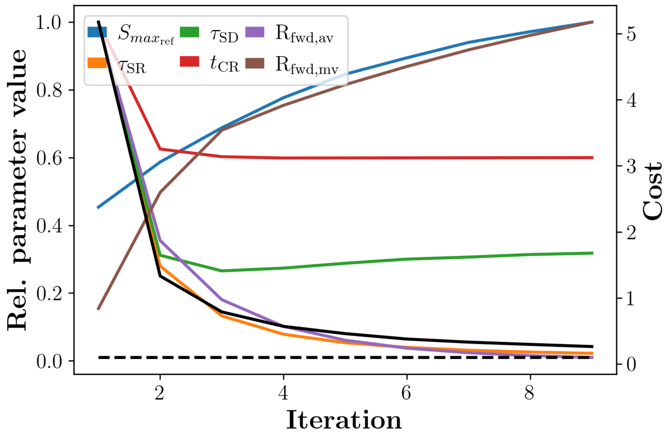

Figure A2. Convergence behaviour of the parameter value and the cost. For each iteration, the parameter values relative to their respective maxima (left y-axis; solid coloured lines) and the cost (right y-axis; solid black line) are plotted. In addition, the convergence threshold (0.1, dotted black line) is given.
